# Systems-level computational modeling demonstrates fuel selection switching in high capacity running and low capacity running rats

**DOI:** 10.1371/journal.pcbi.1005982

**Published:** 2018-02-23

**Authors:** Michael A. Moxley, Kalyan C. Vinnakota, Jason N. Bazil, Nathan R. Qi, Daniel A. Beard

**Affiliations:** 1 Department of Molecular and Integrative Physiology, University of Michigan, Ann Arbor, Michigan, United States of America; 2 Department of Physiology, Michigan State University, East Lansing, MI, United States of America; 3 Department of Internal Medicine, University of Michigan, Ann Arbor, MI, United States of America; University of Groningen, NETHERLANDS

## Abstract

High capacity and low capacity running rats, HCR and LCR respectively, have been bred to represent two extremes of running endurance and have recently demonstrated disparities in fuel usage during transient aerobic exercise. HCR rats can maintain fatty acid (FA) utilization throughout the course of transient aerobic exercise whereas LCR rats rely predominantly on glucose utilization. We hypothesized that the difference between HCR and LCR fuel utilization could be explained by a difference in mitochondrial density. To test this hypothesis and to investigate mechanisms of fuel selection, we used a constraint-based kinetic analysis of whole-body metabolism to analyze transient exercise data from these rats. Our model analysis used a thermodynamically constrained kinetic framework that accounts for glycolysis, the TCA cycle, and mitochondrial FA transport and oxidation. The model can effectively match the observed relative rates of oxidation of glucose versus FA, as a function of ATP demand. In searching for the minimal differences required to explain metabolic function in HCR versus LCR rats, it was determined that the whole-body metabolic phenotype of LCR, compared to the HCR, could be explained by a ~50% reduction in total mitochondrial activity with an additional 5-fold reduction in mitochondrial FA transport activity. Finally, we postulate that over sustained periods of exercise that LCR can partly overcome the initial deficit in FA catabolic activity by upregulating FA transport and/or oxidation processes.

## Author summary

Our bodies consume carbohydrates, fats, and amino acids as fuels, utilizing various catabolic pathways to transfer the energy required for normal physiological functions. The way these pathways function can have an important impact on overall health. While most catabolic pathways are known, we are still striving to understand how these pathways interact, are controlled, and change during exercise and in disease. Here, we have used computer modeling as a tool to understand fuel utilization differences during exercise for two animal models. High capacity running rats (HCR) were used as a healthy, fit cohort, and low capacity running rats (LCR) were used as a sedentary and disease-prone cohort. Our computer model results show that the HCRs are superior at fat utilization compared to LCRs because of their increased ability to transport and catabolize fatty acids. We postulate that these differences depend on exercise intensity and duration, such that longer acclimation periods may minimize fuel utilization differences between these rats.

## Introduction

Fuel selection, the balancing of glucose, fatty acids, and amino acid utilization to match the ATP demand, is a hallmark of healthy metabolism [1]. It has been shown that endurance trained individuals can better maintain the oxidation of fatty acid (FA) throughout mild to moderate intensity exercise [2–4]. A number of studies have shown that exercise training increases skeletal muscle mitochondrial density [5–7], intramuscular fat storage [8, 9], transport [10], and oxidation [7, 11]. These observations corroborate the idea that endurance athletes are better equipped to handle aerobic exercise because of their increased ability to utilize FA as a fuel source. However, FA utilization may not be as important, for instance with competitive endurance runners [12, 13], since their sustained exercise intensity typically exceeds 60% VO2max, a regime where most individuals have crossed over from dominant FA to carbohydrate utilization [14]. Thus, exercise fuel utilization depends on its intensity (%VO2max). Normalizing for this produces similarities in fuel utilization even among various organisms [15–17]. Yet, there are many studies that have demonstrated differences between subjects, namely exercise trained and untrained individuals [2, 3, 18, 19], even when accounting for normalization (%VO2max).

A popular mechanistic view of fuel selection centers on the Randle cycle [20, 21], which explains the preference for FA by the inhibition of the pyruvate dehydrogenase complex, an enzyme at the interface of glycolysis and the TCA cycle and a gate keeper for aerobic glucose oxidation. Extensions [20] to the Randle cycle have been proposed, such as the inhibition of carnitine palmitoyltransferase-1 by malonyl-CoA [22], to explain how glucose oxidation inhibits fatty acid oxidation (FAO). Although the Randle cycle is proposed to describe the reciprocal relationship between glucose and FAO [23], it has been shown to be too simple to explain the etiology of type 2 diabetes [24, 25] and fuel selection in exercise [17, 26, 27].

High and low capacity running rats [28], (HCR and LCR) are genetically outbred rats that have been bred to model extreme genotypes and phenotypes with regard to exercise performance. The main difference in exercise ability between these rats lies in skeletal muscle [29] substrate selection between glucose and FA metabolism [30]. Recently, it has been shown that HCR can effectively utilize FA during graded treadmill exercise, where LCR largely lack this ability [30]. To explore this phenomenon, we have developed a computer model of whole-body central metabolism that accounts for mass balance and thermodynamic constraints to simulate fuel selection during exercise.

**Fig 1:**
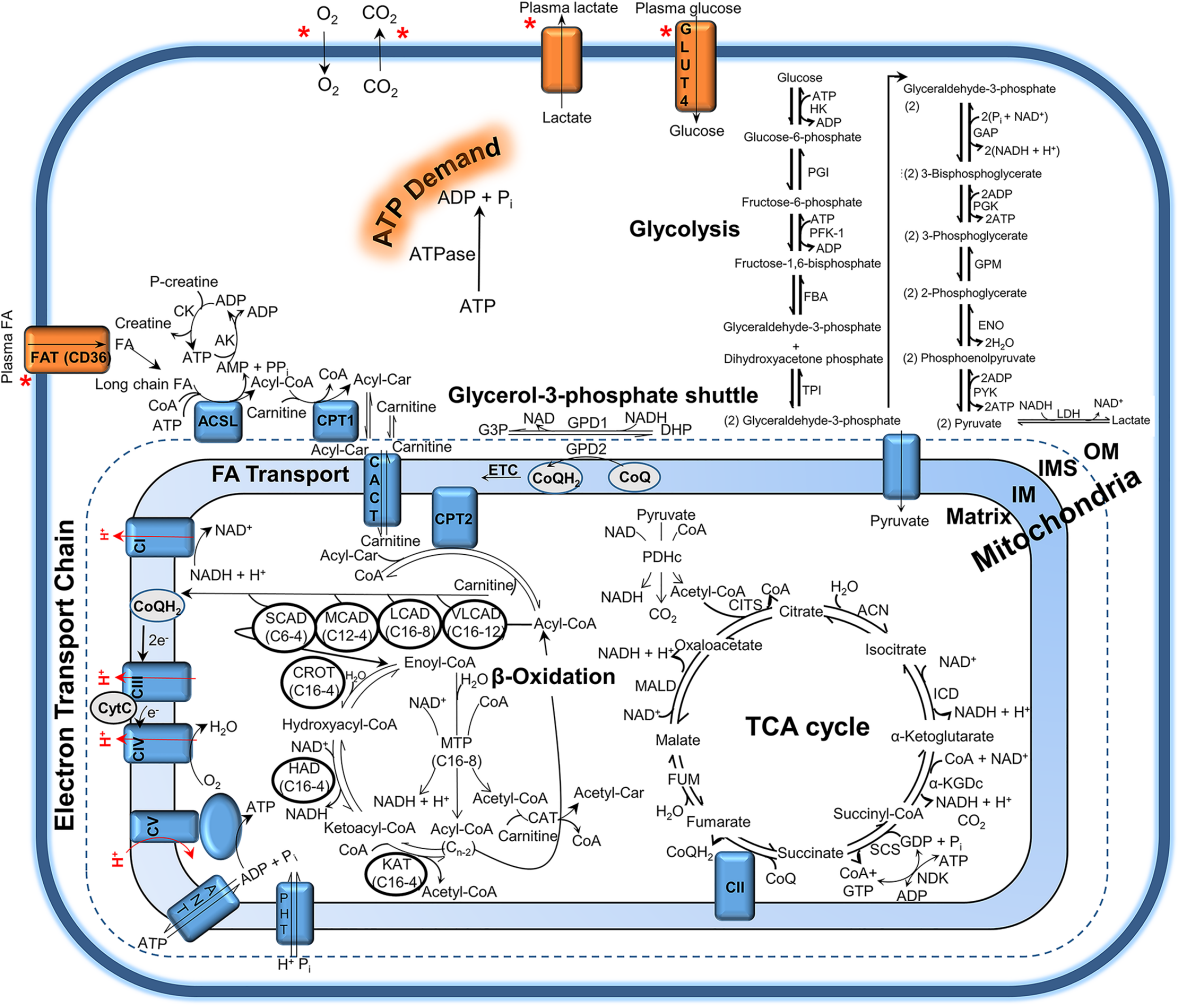
Central pathways of glucose and fatty acid catabolism and transport. Glucose and FA are oxidized via the cytosolic reactions of glycolysis and the mitochondrial reactions of β-oxidation, respectively. The mitochondrial outer membrane (OM), inner membrane space (IMS), inner membrane (IM), and matrix are labeled. Transport fluxes for glucose (GLUT4), FA (CD36), O_2_, CO_2_, and lactate considered by the constraint-based modeling approach are indicated by a red asterisk. Metabolic demand, or ATP demand, in the model is determined by the ATP hydrolysis rate. All model reactions are explicitly defined in S1 Table.

Our model explicitly accounts for all the reactions in glycolysis, the TCA cycle, the mitochondrial electron transport chain, as well as FA transport and beta-oxidation reactions (Fig 1). Overall, the model includes 98 metabolites and 87 reactions (Fig 1), and has been parameterized with HCR and LCR respiration, plasma lactate, and muscle acyl-carnitine concentrations as a function of exercise [30]. Our primary objective was to find the minimal difference between HCR and LCR enzymatic activities required to simulate the apparent fuel selection difference between these animals [30].

Our results show that by minimally decreasing total HCR mitochondrial activity by 30–50% and by further reducing mitochondrial FA transport, we can match the LCR’s attenuated FA utilization activity during transient aerobic exercise. However, when these data are normalized with %VO2max and compared with steady-state data at 75% VO2max there is a major difference in respiratory exchange quotients between LCR transient and steady-state data for the LCR, but not for the HCR. We found that this difference may be explained, by the model, if FA transport and oxidative capacity are much more slowly mobilized upon exercise in the LCR compared to the HCR.

## Results

### HCR and LCR exercise data

HCR and LCR metabolic data were obtained, during a graded treadmill experiment, from a previous publication [30]. In these experiments, HCR (n = 23) and LCR rats (n = 16) were ran on a treadmill with speed increasing every 2 minutes until exhaustion. HCR and LCR rats exhausted, on average, at about 50 and 14 minutes, respectively [30]. During the exercise regimen, O_2_ uptake, CO_2_ output, as well as carbohydrate and FA utilization were estimated [30]. Blood plasma was also sampled for glucose, FA, and lactate [30]. Additionally, gastrocnemius muscle biopsies were taken at 0 and 10 minutes, for both HCR and LCR, and at 45 minutes for HCR only [30]. Muscle biopsies and blood plasma were analyzed by metabolomics and proteomic methods [30].

### HCR and LCR constraint-based modeling

**Fig 2:**
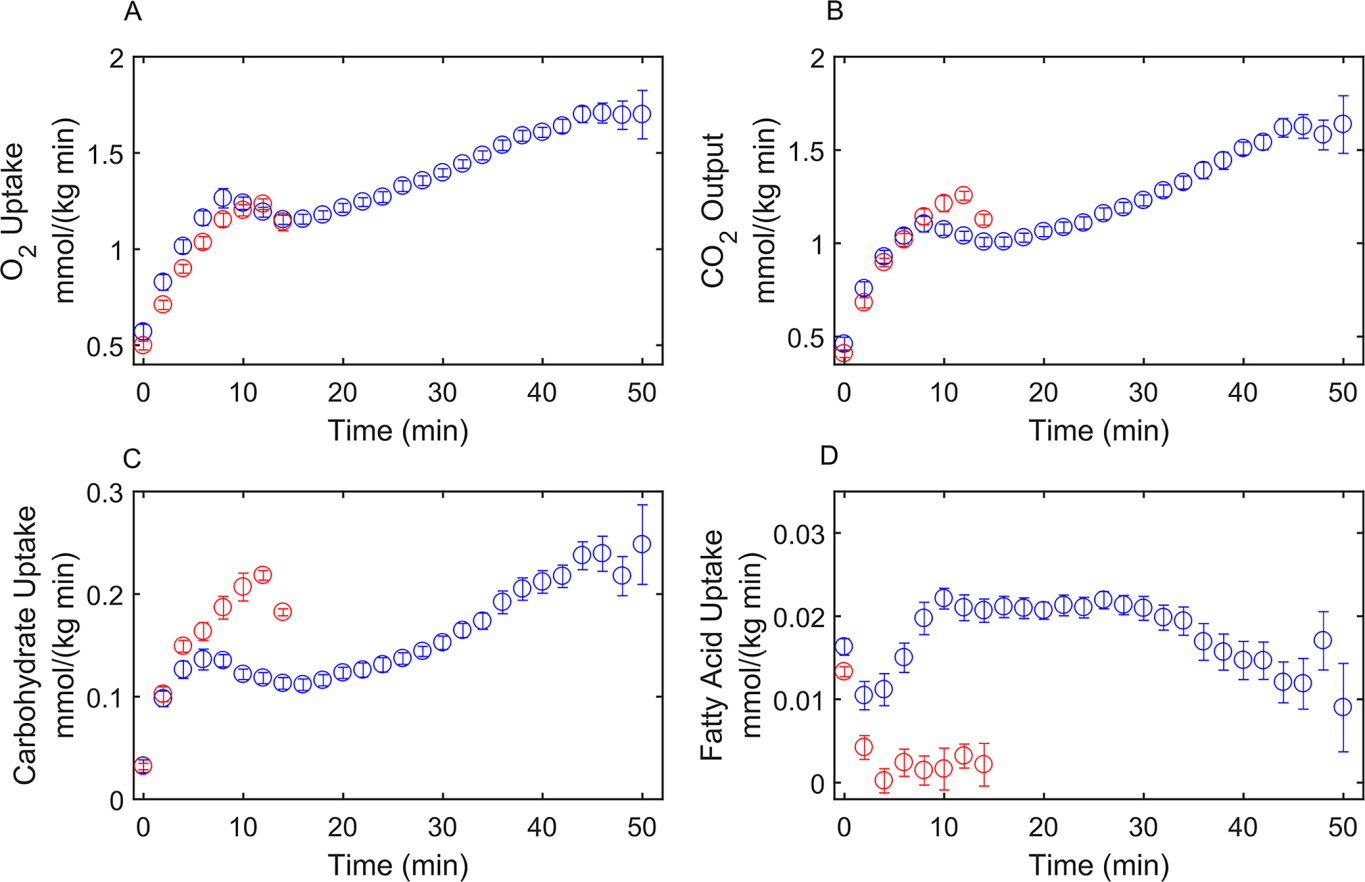
Metabolic transport fluxes for HCR and LCR rats. Transport fluxes were obtained during a previously published graded treadmill experiment [30] and converted to molar quantities. In this experiment, HCR and LCR rats were ran at an increasing treadmill speed (every 2 minutes) until exhaustion. (A) O_2_ uptake fluxes for HCR (blue) and LCR (red) rats. (B) CO_2_ output fluxes for HCR (blue) and LCR (red) rats. (C) Estimated carbohydrate uptake flux for HCR (blue) and LCR (red) rats. (D) Estimated FA uptake fluxes for HCR (blue) and LCR (red) rats.

HCR and LCR O_2_ and CO_2_ flux data (Fig 2A and 2B), and carbohydrate and FA flux data (Fig 2C and 2D), estimated from indirect calorimetry, were previously collected [30] during a graded treadmill exercise protocol (described above). These data (Fig 2) were used as transport flux inputs for a constraint-based [31, 32] calculation to compute all 87 internal reaction fluxes of the metabolic system shown in Fig 1 for HCR and LCR data (reactions shown in S1 Table). Transport fluxes (Fig 2) at each time point for HCR and LCR were used to solve for internal fluxes ($\bar{J}$) (Eq 1, Methods), while maximizing mitochondrial ATP production as an objective function and applying *ab initio* thermodynamic cycle constraints for the network [33].

**Fig 3:**
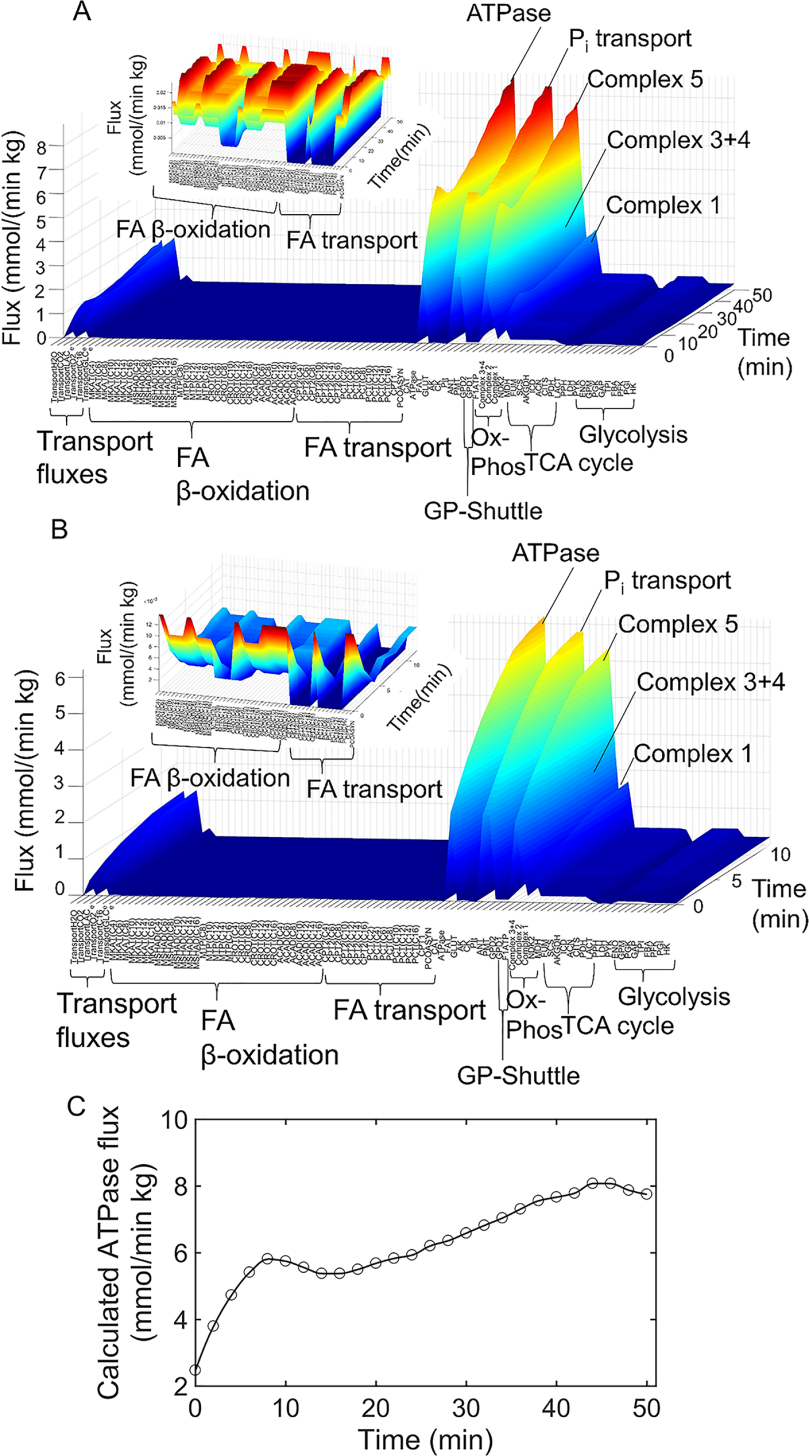
HCR and LCR metabolic constraint-based solutions. (A) HCR constraint-based solution: Eq 1 (in Methods) was solved at each time point for internal reaction fluxes using given carbohydrate, FA, O_2_, and CO_2_ transport fluxes. Transport fluxes were derived from HCR O_2_ and CO_2_ flux data [30] shown in Fig 2. (Inset) Zoomed in view of HCR mitochondrial FA transport and beta-oxidation enzyme fluxes. (B) LCR constraint-based solution: Eq 1 (in Methods) was solved at each time point for internal reaction fluxes using given carbohydrate, FA, O_2_, and CO_2_ fluxes [30] shown in Fig 2. (Inset) Zoomed in view of LCR mitochondrial FA transport and beta-oxidation enzyme fluxes. (C) The ATPase (ATP → ADP + Pi) flux (circles) from the HCR constraint-based solution in panel A was used to estimate the ATPase rate with time for subsequent simulations.

Initial FA, was simplified to be only palmitate (C16 acyl chain length), and glucose was the only carbohydrate considered. The computed 87 internal reaction fluxes, with time, are shown for HCR (Fig 3A) and LCR (Fig 3B). The reaction fluxes are annotated by an enzyme identifier (Shown in S1 Table), and grouped by major pathways shown in Fig 1. The constraint-based solution demonstrates the incline of metabolic flux with exercise for HCR (Fig 3A) and LCR (Fig 3B), most consistently through the TCA cycle, glycolytic pathway, and bioenergetic reactions. The solution also demonstrates the attenuated flux through the FAO pathway for LCR (inset Fig 3B) relative to HCR (inset Fig 3A). The constraint-based solution is a first approximation (quasi-steady state) that allows an estimation of internal fluxes and a starting point for parameterization of a kinetic modelling approach. The fluxes represent whole-body rates, measured in units of moles per unit time per body weight. The ATPase rate was used from this constraint-based solution at each time point (Fig 3C) to simulate the work load, or ATP demand, in HCR and LCR in the subsequently described kinetic model.

To test the stability of the HCR and LCR solutions (Fig 3), we determined how uncertainty in the measured boundary fluxes translates into uncertainty in the estimated fluxes. Results from these analyses are summarized in S1–S3 Figs. The maximum and minimum solutions for HCR (S1 Fig) and LCR (S2 Fig), at each time point, differ by only a small margin.

### HCR and LCR dynamic modeling

**Fig 4:**
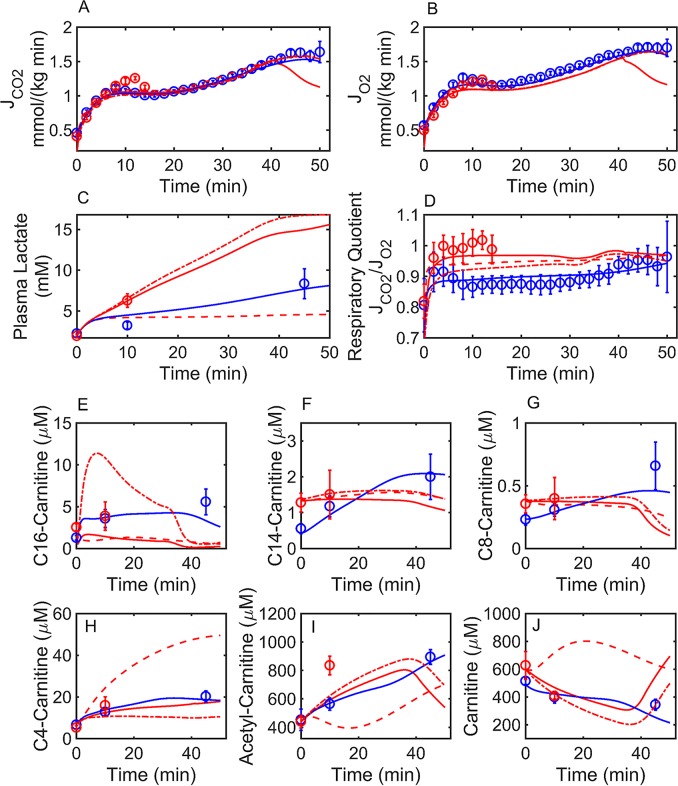
Simulation of HCR and minimal simulation of LCR rat exercise data. HCR (blue circles) and LCR (red circles) rat data were collected previously [30] during a graded treadmill experiment. Major pathways (87 reactions) of glucose, FA transport, oxidation, and bioenergetics (Fig 1) were simulated using an ordinary differential equation system (98 state variables). Enzyme activities (*X*, Eq 4) were adjusted to fit the HCR data (blue circles), while a change in HCR enzyme activities were used to fit the LCR data (red circles). Simulations are represented by lines for HCR (blue) and LCR (red). LCR: solid lines were simulated by decreasing HCR total mitochondrial and FAO activities (error function value = 2.68), red dash-dot lines were simulated by decreasing total HCR mitochondrial enzyme activities only (error function value = 2.89), and dashed lines were simulated by decreasing HCR FAO enzyme activities only (error function value = 4.03). Total acyl-carnitine concentrations shown in panels (E-J) were derived from HCR and LCR gastrocnemius muscle [30]. Error bars represent standard error of the mean, while error bars in panel D were calculated from the propagation of error using errors in JCO_2_ and JO_2_ fluxes shown in panel A and B, respectively. (A) HCR and LCR rat carbon dioxide flux (JCO_2_). (B) HCR and LCR rat molecular oxygen flux (JO_2_). (C) Plasma lactate from HCR and LCR. (D) Respiratory quotient (JCO2/JO2) for HCR and LCR. (E) Total C16-carnitine muscle concentration for HCR and LCR rats. (F) Total C14-carnitine muscle concentration for HCR and LCR rats. (G) Total C8-carnitine muscle concentration for HCR and LCR rats. (H) Total C4-carnitine muscle concentration for HCR and LCR rats. (I) Total Acetyl-carnitine muscle concentration for HCR and LCR. (J) Total Carnitine muscle concentration for HCR and LCR rats.

A kinetic model for simulating HCR and LCR fuel selection was initially identified using fluxes from the constraint-based solution (Fig 3) and using Eq 4 (Methods) to assign value for the enzyme activities (*X*). Furthermore, ATP demand was simulated based on the interpolated rate from the constraint-based solution (Fig 3C). Initial concentrations for 98 metabolites were obtained by a Monte Carlo method that randomly searched for a concentration vector satisfying the thermodynamic constraint of Eq 2 (Methods). The directions of the fluxes in Eq 2 (Methods) were determined from the constraint-based solution at rest (time = 0). Reasonable concentration bounds determined from the literature were imposed on the Monte Carlo search (Reported in S2 Table as lower (LB) and upper bounds (UB)). Finally, the activities were adjusted to fit the HCR data in Fig 4, as described below. The initial concentration vector used to simulate the data in Fig 4 is reported in S2 Table.

HCR CO_2_ (Fig 4A) and O_2_ (Fig 4B) fluxes (J_CO2_ and J_O2_), plasma lactate (Fig 4C), respiratory quotient (RQ; Fig 4D), and muscle acyl-carnitine profiles (Fig 4E–4J) (all data derived from [30]) were used to parameterize the model by least-squares minimization. With a total of 86 adjustable parameters, the solution illustrated in Fig 4 is not unique. Uncertainty of parameter values and their predictions were assessed below.

After fitting the HCR data (Fig 4; blue), we tested the hypothesis (Hypothesis 1) that the metabolic data can be simulated by invoking only a difference in mitochondrial density between HCR and LCR (Fig 4; red dash-dot lines). This hypothesis is derived from the observations that trained individuals have a higher proportion of mitochondria per muscle mass than untrained individuals, and this difference has been observed in both HCR and LCR rats [6, 34].

Simulation of hypothesis 1 significantly deviated from the RQ data, where the error between the model and the data were quantitated by the sum of squared error function values defined in Eq 7 (Fig 4D; red dash-dot lines; error function value = 2.89). Thus, decreasing total HCR mitochondrial activity only (optimal near a 50% decrease) could not sufficiently switch HCR to utilize glucose in an aerobic manner, as demonstrated by LCR data (Fig 4D; red dash-dot lines). However, this hypothesis does cause the model to switch to utilize glucose in an anaerobic manner (Fig 4C; red dash-dot lines). Decreasing total HCR mitochondrial activity beyond 50% did not improve fitting to the LCR data.

Second, we tested the hypothesis that HCR and LCR only differ in total FA utilization (Fig 4; dashed lines). We found that this hypothesis (Hypothesis 2) was also somewhat inconsistent with the data (error function value = 4.03). Decreasing only FAO enzyme activities (optimal decrease around 10-fold) matches the RQ data for LCR (Fig 4D; dashed lines) better than hypothesis 1, indicative of increasing the aerobic oxidation of glucose. But decreasing FAO enzymes only was unable to match the observed increase in anaerobic glucose utilization (Fig 4C; dashed lines) and acetyl-carnitine concentrations with exercise (Fig 4I; dashed lines).

**Fig 5:**
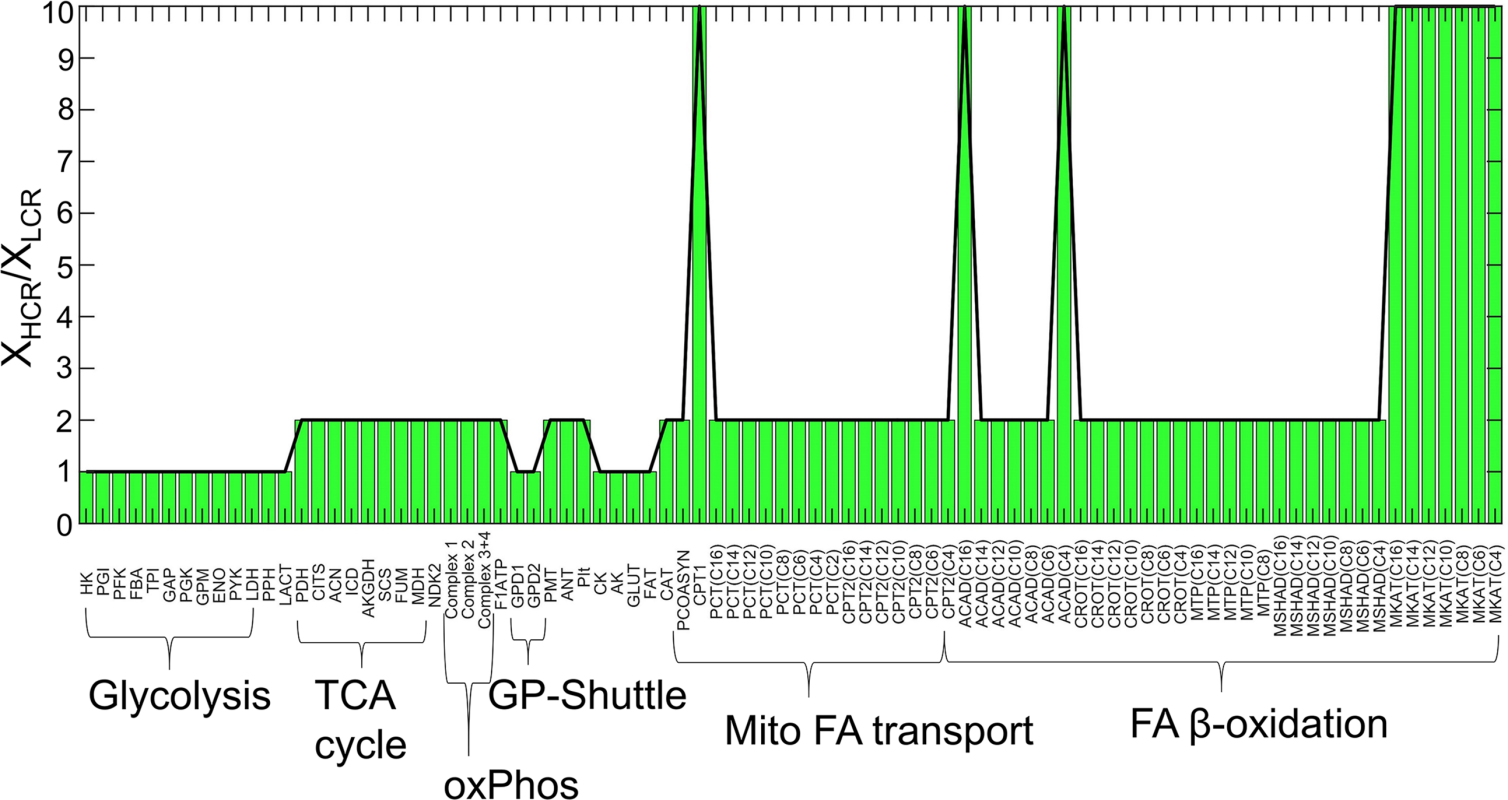
Ratios of HCR/LCR enzyme activities for minimal LCR simulation. The enzyme activities used to fit the HCR and LCR data in Fig 4 were used to create the ratio X_HCR_/X_LCR_. Enzyme activities are labeled by shortened identifiers, described in S1 Table, and further grouped with brackets into major pathways defined in Fig 1.

These observations led us to alter both total mitochondrial enzyme and FAO activities to achieve a minimal difference between HCR and LCR enzyme activities (Fig 4; solid lines; error function value = 2.68). By minimal, we are referring to the minimal number of enzyme activity differences between model parameterizations representing the LCR versus the HCR data. To be clear, when both mitochondrial enzyme activities and FAO enzyme activities were decreased this means that after applying, for example, a 50% decrease in all mitochondrial enzymes an additional decrease in FAO enzyme activities was applied to help fit the LCR data. This additional difference, between FAO enzymes and all other mitochondrial enzymes, can be seen in Fig 5, which is discussed below. We found that decreasing FA transport enzyme activities was the most effective way to switch fuel usage from a more FAO dominant mode to a more glucose oxidative mode. Decreasing total mitochondrial enzyme activity between 30% and 50% was also required to best-fit the data.

The predicted ratios of HCR and LCR enzyme activities (X_HCR_/X_LCR_) are shown in Fig 5 for the minimal best-fit parameter set of the LCR data (Fig 4; red solid lines). The minimal best-fit parameter set for LCR has decreased all mitochondrial enzyme activities by 50% and additionally decreased several FAO related enzyme activities relative to HCR.

**Fig 6:**
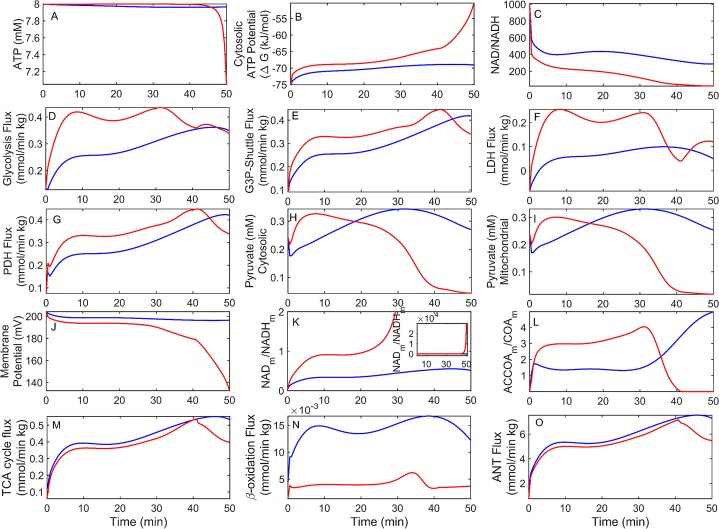
Simulation of HCR and LCR metabolism during exercise. In all panels, HCR and LCR simulations are represented by blue and red lines, respectively. Simulations were performed using the minimal difference parameter set between HCR and LCR (Figs 4 and 5). (A) Cytosolic ATP with time. (B) Gibbs free energy potential (Eq 2, Methods) for cytosolic ATP hydrolysis with time. (C) Cytosolic ratio of oxidized (NAD^+^) over reduced (NADH) NAD with time. (D) Time-dependent average flux from glycolytic enzymes. (E) Time-dependent average flux of glycerol-3-phosphate (G3P) shuttle enzymes. (F) Lactate dehydrogenase flux with time. (G) Pyruvate dehydrogenase (PDH) complex flux with time. (H) Cytosolic pyruvate with time. (I) Mitochondrial pyruvate with time. (J) Time-dependent mitochondrial membrane potential. (K) Mitochondrial NAD ratio for oxidized (NAD^+^) over reduced (NADH) NAD with time. Inset is a zoomed-out view of the main panel. (L) Mitochondrial Acetyl-CoA to CoA ratio with time. (M) Time-dependent average flux of TCA cycle enzymes. (N) Time-dependent average flux of FA β-oxidation enzymes. (O) Adenine nucleotide transporter (ANT) flux with time.

The model was used to probe dynamic variables during exercise that are difficult to measure (Fig 6). The model demonstrates constant cytosolic ATP (Fig 6A; blue) and decreasing (more positive) cytosolic ATP potential (Fig 6B), consistent with previous *in vivo* measurements during prolonged exercise [35]. Interestingly, the model simulation for LCR eventually fails because it is unable to meet the ATP demand (Fig 6A; red). However, this phenomenon occurs long after LCR have exhausted in the experimental data (Fig 4). The model demonstrates LCR’s increased flux through aerobic glucose oxidation (Fig 6D and 6E) and anaerobic or glycolytic flux (Fig 6F), but has reduced FAO (Fig 6N) flux compared to HCR. The model also demonstrates that as work increases the NAD pool becomes more reduced (Fig 6C) in the cytosolic fraction whereas the converse occurs in the mitochondrial fraction (Fig 6K). The model predicts LCR is eventually unable to maintain the mitochondrial membrane potential (Fig 6J) due to the failure of aerobic glucose oxidation.

One of the earliest indicators of failure in LCR is the drop in the acetyl-CoA/CoA ratio (Fig 6L), where acetyl-CoA decreases and CoA increases. The cause for the eventual crashing is that the cytosolic NAD pool becomes overly reduced (Fig 6C) slowing down glycolysis (Fig 6D), subsequent production of pyruvate (Fig 6H and 6I), and acetyl-CoA feeding into the TCA cycle (Fig 6M). This leads to decreased mitochondrial ATP output (Fig 6O), which is unable to meet the ATP demand (Fig 6A). Although β-oxidation decreases (Fig 6N) at about 35 minutes in the simulation it does not crash like glycolysis. These simulation results are generally consistent when using other enzyme activities that also fit the data, discussed below.

Predicted metabolite concentrations for HCR and LCR are available in S3 and S4 Tables for HCR and LCR, respectively. These predicted metabolite concentrations were generated based on the activities used to produce Fig 6. Ratios of the simulated metabolite concentrations as a function of time, and exercise, demonstrate further predicted differences (S3 and S4 Figs) between HCR and LCR. These figures predict higher concentrations of initial FAO substrates for HCR (S3 Fig) compared to LCR, whereas LCR has accumulated more FAO end products (S4 Fig). This figure also demonstrates the model prediction that HCR will accumulate AMP and PPi (S4A Fig), a consequence of HCR’s relative increased FAO flux compared to LCR.

### Parameter confidence and sensitivity

**Fig 7:**
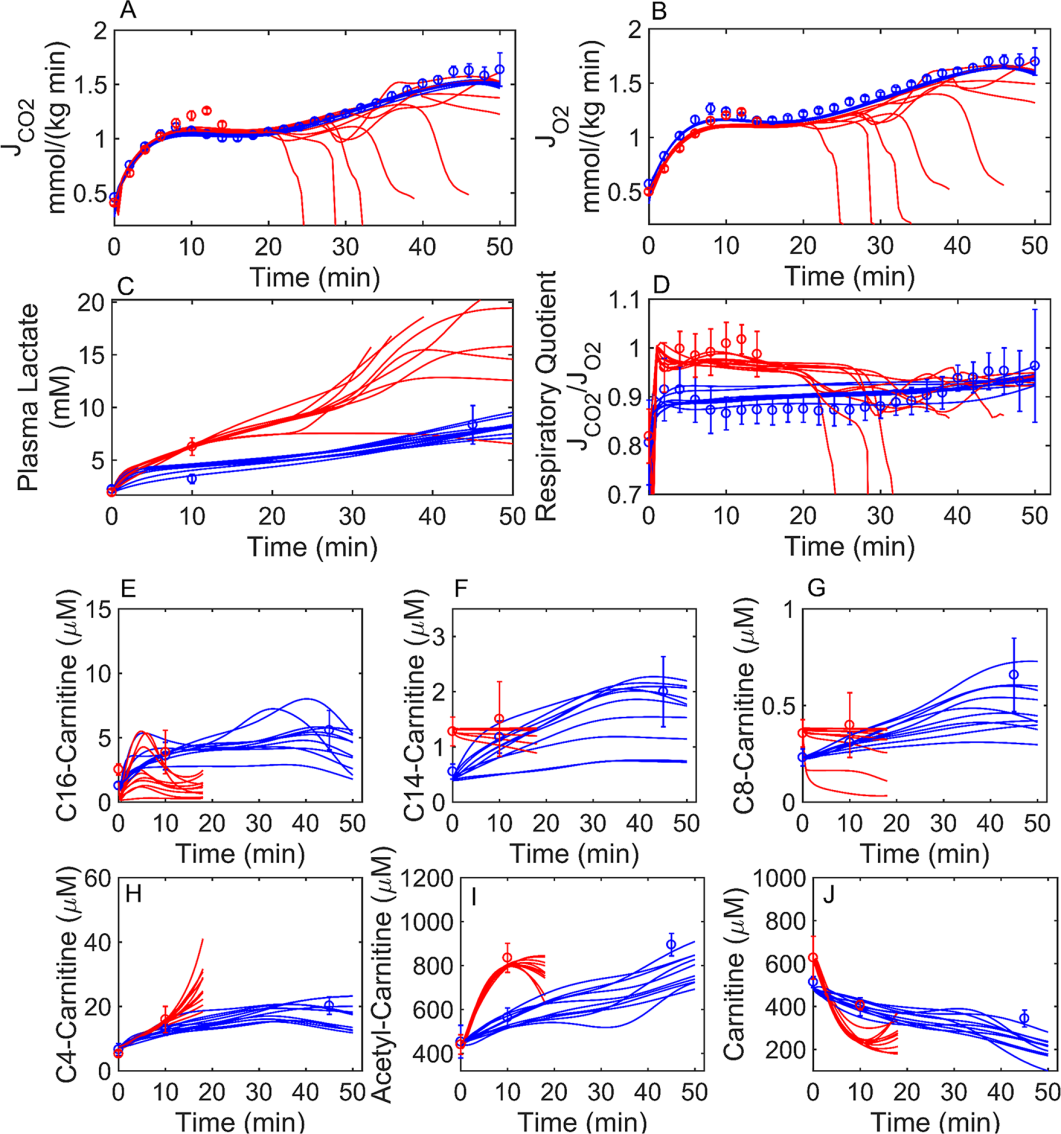
Simulation families of HCR and LCR data. HCR (blue circles) and LCR (red circles) rat exercise data [30] are as described in Fig 4. Several independent attempts were made to simulate the HCR and LCR data by independently adjusting the enzyme activities yielding 10 families of fits. All simulations are depicted by lines of the corresponding color (HCR (blue) and LCR (red)). Error bars represent standard error of the mean, while error bars in panel D were calculated from the propagation of error formula using errors in JCO_2_ and JO_2_ fluxes shown in panel A and B, respectively. LCR simulations (red lines) are truncated at 18 minutes in panels E-J for clarity. (A) HCR and LCR rat carbon dioxide flux (JCO_2_). (B) HCR and LCR rat molecular oxygen flux (JO_2_). (C) Plasma lactate from HCR and LCR. (D) Respiratory quotient (JCO2/JO2) for HCR and LCR. (E) Total C16-carnitine muscle concentration for HCR and LCR rats. (F) Total C14-carnitine muscle concentration for HCR and LCR rats. (G) Total C8-carnitine muscle concentration for HCR and LCR rats. (H) Total C4-carnitine muscle concentration for HCR and LCR rats. (I) Total acetyl-carnitine muscle concentration for HCR and LCR rats. (J) Total carnitine muscle concentration for HCR and LCR rats.

To evaluate the robustness of our results, we obtained an ensemble of independent parameter sets capable of fitting the data. To achieve this, we randomly perturbed the parameters from an initial fit to the HCR data (Fig 4; blue) and fed them into a simulated annealing algorithm followed by a local optimization algorithm. By this strategy, we obtained 10 different fits to the HCR data (Fig 7; blue). Each of these HCR parameter sets were then used to obtain 10 minimal fits to LCR data by only changing total mitochondrial activities and the select activities shown in Fig 5. The minimal changes made to these HCR parameter sets to fit LCR data are all similar to those shown in Fig 5.

HCR parameters were also used as initial starting points to find 10 non-minimal fits to the LCR data (Fig 7; red). That is, we allowed all parameters to vary independently to match the LCR data. Like the minimal best-fit LCR simulation in Fig 4, we observed that 7 out of these 10 LCR simulations eventually failed because they were unable to meet the ATP demand by relying on mostly glucose oxidation for an extended period of exercise. The difference, however, between the parameter sets used to simulate the LCR data in Fig 4 and in Fig 7, is that the latter parameter sets were generated by allowing all enzyme activities to change to fit the LCR data. In other words, the LCR enzyme activities used to simulate LCR in Fig 7 do not reflect a minimal difference relative to HCR enzyme activities.

**Fig 8:**
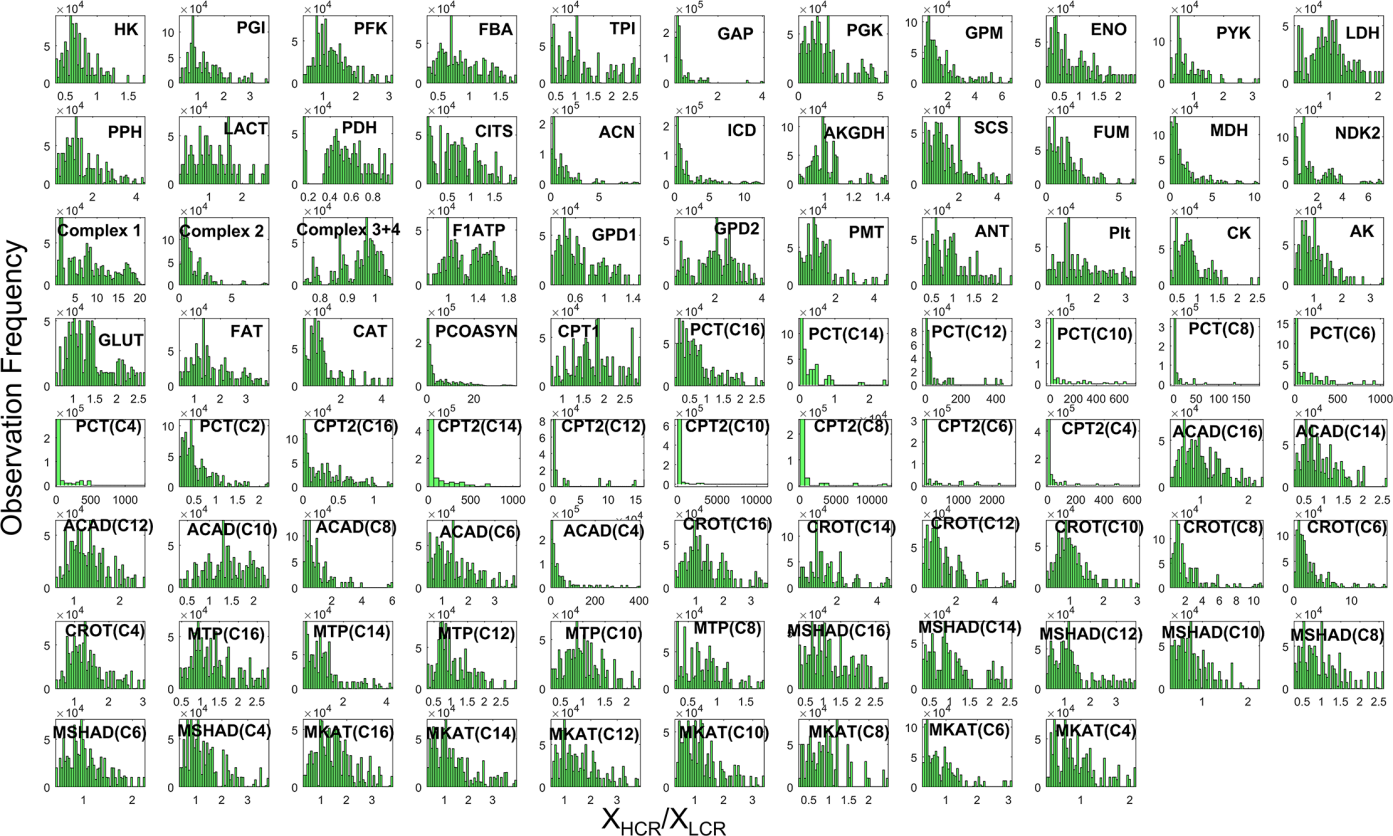
Histograms of HCR and LCR enzyme activity ratios (X_HCR_/X_LCR_) from multiple parameter sets. A 10^3^ parameter sets, each composed of 86 enzyme activities, individually fitted to HCR and LCR data were used to obtain a multitude of enzyme activity ratios (X_HCR_/X_LCR_). All combinations of each of the 10^3^ parameter sets for HCR and LCR were used yielding 10^6^ ratios for each of the 86 enzyme activities. Each histogram panel in the figure represents one of the 86 enzyme activity ratios and are annotated per the shortened enzyme identifier defined in S1 Table. In each histogram panel, the y-axis represents the frequency of ratio (X_HCR_/X_LCR_) occurrence and the x-axis represents the value of the ratio.

To obtain a distribution of activity ratios (X_HCR_/X_LCR_), such as that shown in Fig 5 for a minimal difference, we randomly perturbed each of the 10 parameter sets for HCR (Fig 7; blue) and LCR (Fig 7; red) until we obtained 10^3^ parameter sets capable of fitting each data set. We then took the ratio of all possible combinations of parameter sets between HCR and LCR yielding 10^6^ ratios (Fig 8) for each enzyme activity. This demonstrates that the main difference between HCR and LCR enzyme activities lies in mitochondrial acyl-CoA/acyl-carnitine transport. The widest range in parameter differences between HCR and LCR is in the palmitoyl-CoA translocase (PCT) and carnitine palmitoyltransferase-2 (CPT2) activities. However, the output of the model is not particularly sensitive to these activities.

Although a number of enzyme activities (model adjustable parameters) tend to fall within a relatively small range (Fig 8) to fit the data (Fig 7) this does not mean that we have identified absolute enzyme activities because of the correlations that exist in parameter space and because of the simplified nature of the kinetic model. Thus, one should not assign strict value to these enzyme activities, but understand that simulating LCR data from a HCR model starting point can generally be achieved by minimally altering FAO and FA transport enzyme activities with some additional decrease in total mitochondrial enzyme activity.

**Fig 9:**
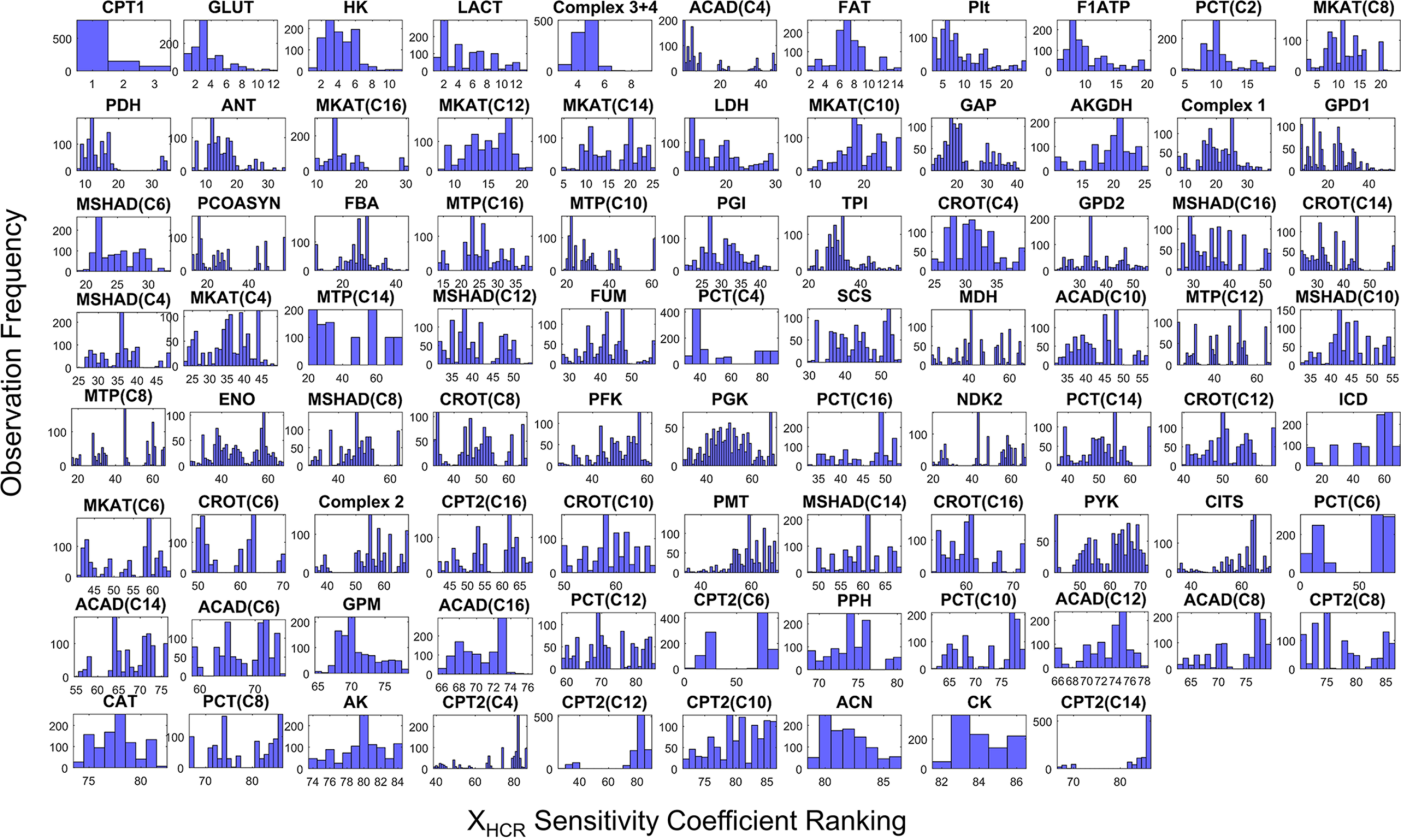
Histograms of HCR enzyme activity (X_HCR_) sensitivities to fuel selection ranked from multiple HCR parameter sets. A 10^3^ parameter sets, each composed of 86 enzyme activities, individually fitted to HCR were used to obtain 10^3^ sensitivity coefficients for each activity. Each histogram panel in the figure represents a distribution of one of the 86 enzyme activity sensitivity coefficients. Each panel is annotated per the shortened enzyme identifier defined in S1 Table. In each histogram panel, the y-axis represents the observed occurrence, or frequency, of a sensitivity coefficient and the x-axis represents the rank, or order of sensitivity coefficient magnitude out of 86. For instance, rank 1 is the most sensitive activity (X_HCR_) whereas as rank 86 is the least sensitive activity. Histogram panels are ordered per their median rank value from top left to bottom right in the figure. Thus, the most sensitive activities are ordered from top left to bottom right in the figure.

**Fig 10:**
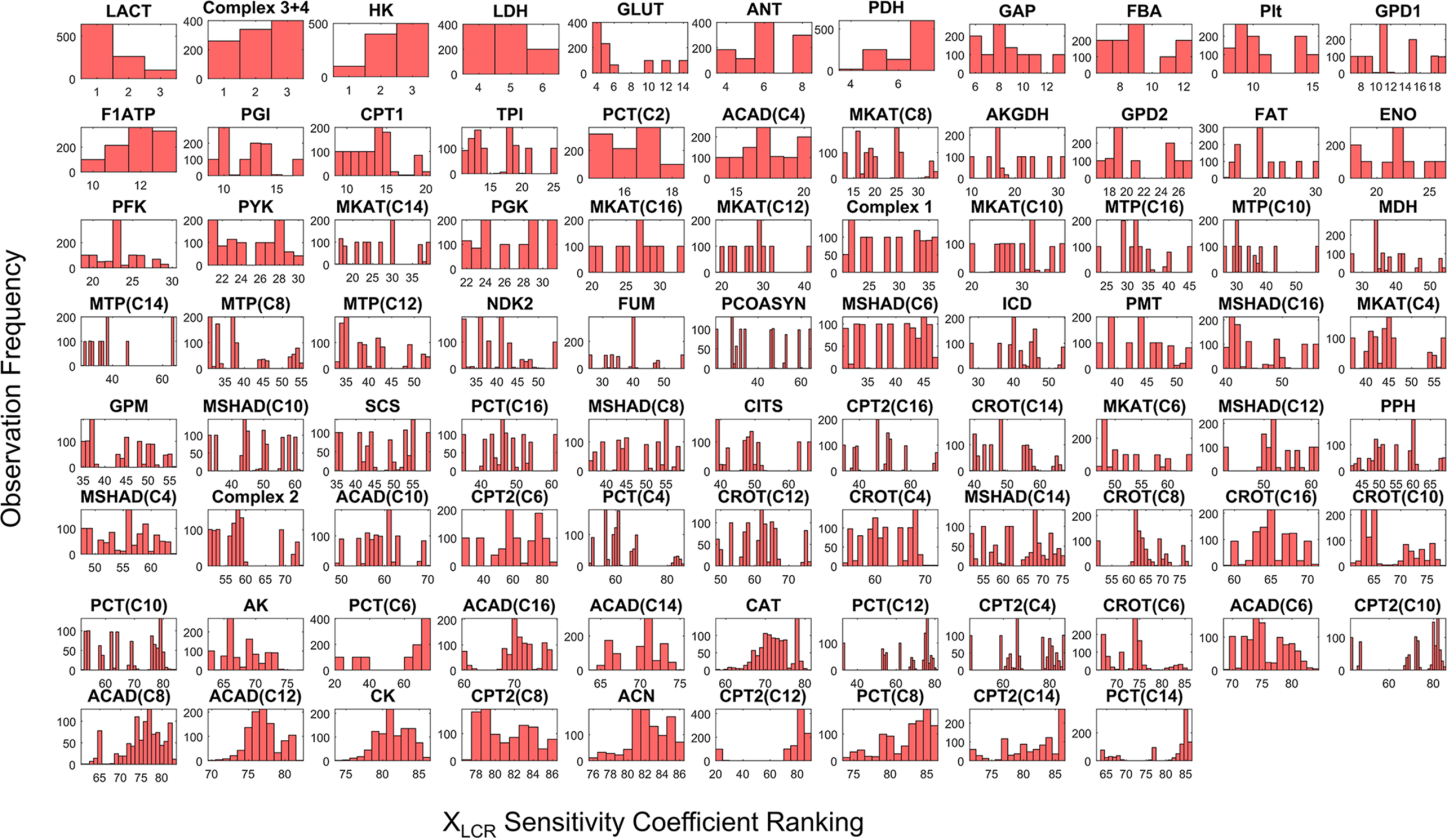
Histograms of LCR enzyme activity (X_LCR_) sensitivities to fuel selection ranked from multiple LCR parameter sets. A 10^3^ parameter sets, each composed of 86 enzyme activities, individually fitted to LCR were used to obtain 10^3^ sensitivity coefficients for each activity. Each histogram panel in the figure represents a distribution of one of the 86 enzyme activity sensitivity coefficients. Each panel is annotated per the shortened enzyme identifier defined in S1 Table. In each histogram panel, the y-axis represents the observed occurrence, or frequency, of a sensitivity coefficient and the x-axis represents the rank, or order of sensitivity coefficient magnitude out of 86. For instance, rank 1 is the most sensitive activity (X_LCR_) whereas as rank 86 is the least sensitive activity. Histogram panels are ordered per their median rank value from top left to bottom right in the figure. Thus, the most sensitive activities are ordered from top left to bottom right in the figure.

In order to determine what enzyme activities are most important to fuel selection in exercise, we calculated sensitivity coefficients for each of the enzyme activities for HCR and LCR for all 10^3^ parameter sets with respect to the simulation of the respiratory quotient (J_CO2_/J_O2_). The respiratory quotient is typically considered to be a good indicator of fuel selection based on stoichiometric utilization of O_2_ and production of CO_2_ for different substrates. The results of this calculation yielding 10^3^ sensitivity coefficients for HCR (Fig 9) and LCR (Fig 10) are displayed in histograms and ordered by high to low sensitivity in the figure. This calculation revealed carnitine palmitoyltransferase-1 (CPT1) activity to be the most sensitive with HCR (Fig 9), while lactate transport (LACT) was the most sensitive with LCR (Fig 10).

Exercise fuel selection in HCR and LCR were also both highly sensitive to glucose transport (GLUT), hexokinase (HK), and the combination of complex 3 and 4 (Complex 3+4) activity (Figs 9 and 10). The main difference between HCR and LCR with respect to fuel selection enzyme sensitivity was with FA utilization enzymes, such that HCR was sensitive to these activities and LCR was not (Figs 9 and 10). Thus, HCR is predicted to have more control of FA utilization relative to LCR, FA transport in particular.

**Fig 11:**
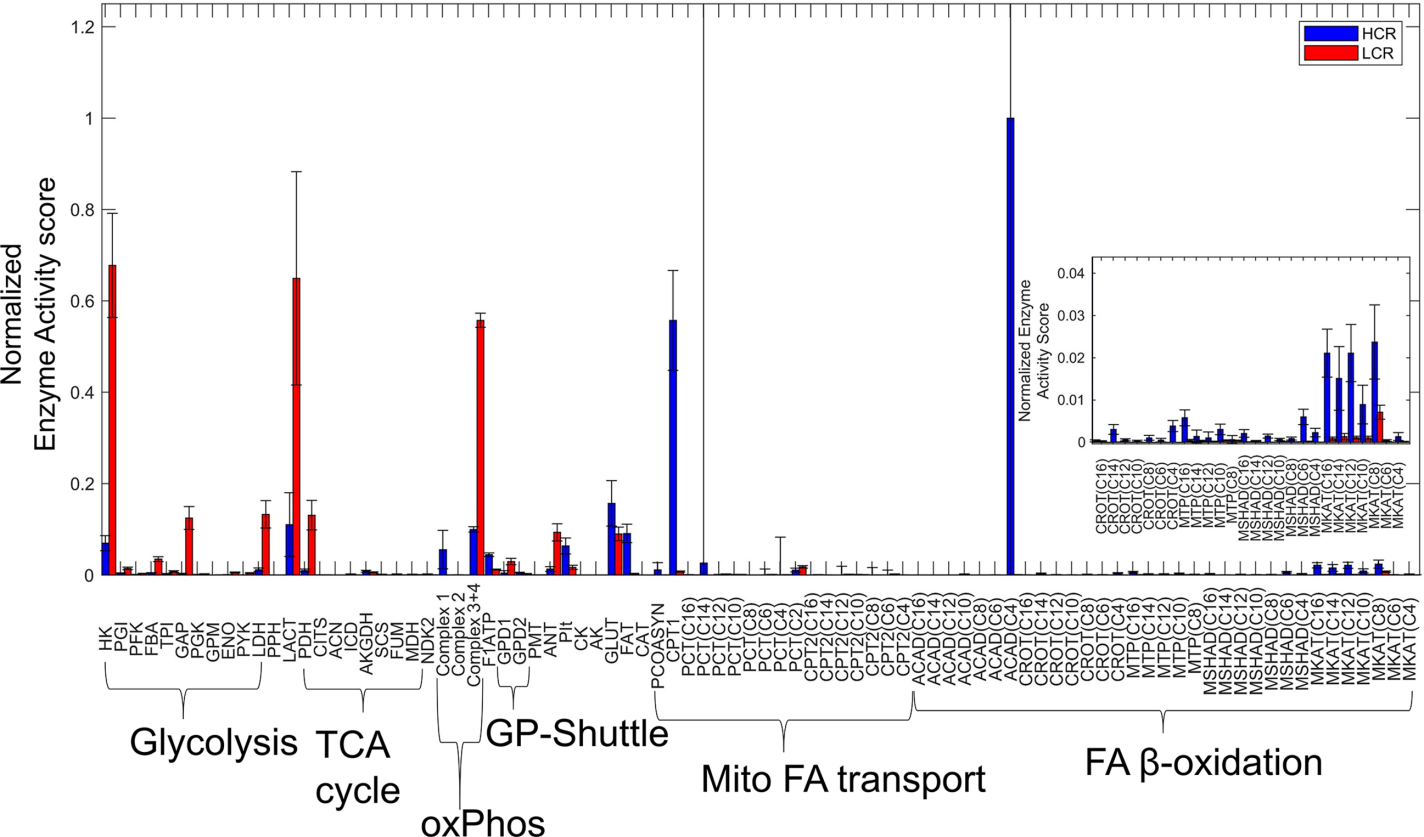
Median HCR and LCR normalized enzyme activity scores. HCR and LCR enzyme activities, each from 10^3^ parameter sets, and their corresponding sensitivity coefficients, were used to calculate a median enzyme activity score to assess each enzyme’s importance in fuel selection. HCR scores are depicted by blue bars and LCR by red bars. Enzyme activity scores are labeled by shortened identifiers, described in S1 Table, and further grouped with brackets into major pathways defined in Fig 1. (Inset) Zoomed in view of certain beta-oxidation enzyme activity scores. Error bars represent standard deviations.

To help combine the information collected by computing numerous enzyme activity ratios (Fig 8) and sensitivity coefficients (Figs 9 and 10), we used a simple expression (Eq 6, Methods) to calculate a median score for the relative importance of each enzyme activity (Fig 11). The purpose of this score was to assess the major differences between HCR and LCR enzyme exercise activities regarding fuel selection, while also accounting for their sensitivity. This calculation is based on the non-minimal fitting of LCR data (Fig 7).

The enzyme score calculation reveals higher importance for glycolytic enzymes for LCR and FA catabolic enzymes for HCR, although our minimal approach to fitting LCR data (Fig 4) showed that glycolytic enzyme activities do not have to change (Fig 5) to fit this data. Interestingly, the importance of CPT1 and ACAD4 was also revealed in this calculation, which agrees with conclusions drawn from the minimal approach to fitting LCR data (compare Fig 5 and Fig 11).

#### Comparison of HCR and LCR transient and steady-state normalized fuel selection and FA utilization capacity

**Fig 12:**
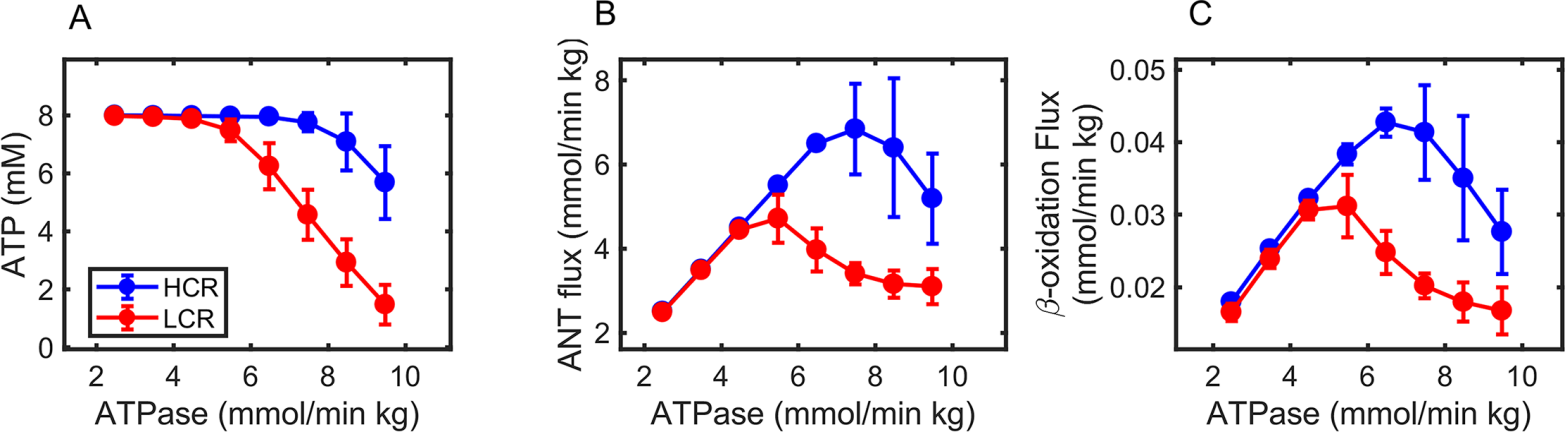
Simulated estimation of HCR and LCR fatty acid oxidation capacity. To theoretically assess FA utilization capacity in HCR (blue) and LCR (red), glycolytic enzyme activities were decreased by several orders of magnitude to eliminate ATP synthesis from glucose. (A) Average ATP concentration at increasing ATPase rates. (B) Average mitochondrial adenine nucleotide transporter (ANT) flux at increasing ATPase rates. (C) Average of β-oxidation enzyme fluxes at increasing ATPase rates. The error bars represent standard deviations from 10 families of HCR and LCR activity parameter sets (Fig 7).

To theoretically estimate the FA utilization capacity of HCR and LCR, we decreased glycolytic activities by several orders of magnitude until no appreciable glycolytic flux was observed. Without the ability to synthesize ATP via glycolysis, and eliminating ATP buffering by creatine kinase, FA utilization is the only means to sustain ATP synthesis in our model. After decreasing these activities, an increasing linear ATPase rate was imposed until the ATP synthesis capacity was overwhelmed by ATP demand, at which point ATP concentration collapses (Fig 12A). With glucose oxidation effectively knocked out, the mitochondrial adenine nucleotide transporter (ANT) flux equals the ATP synthesis flux from FAO (Fig 12B). These calculations revealed, based on 10 parameter sets for HCR and LCR, that the FAO capacity is 30% lower in LCR than HCR (Fig 12C).

**Fig 13:**
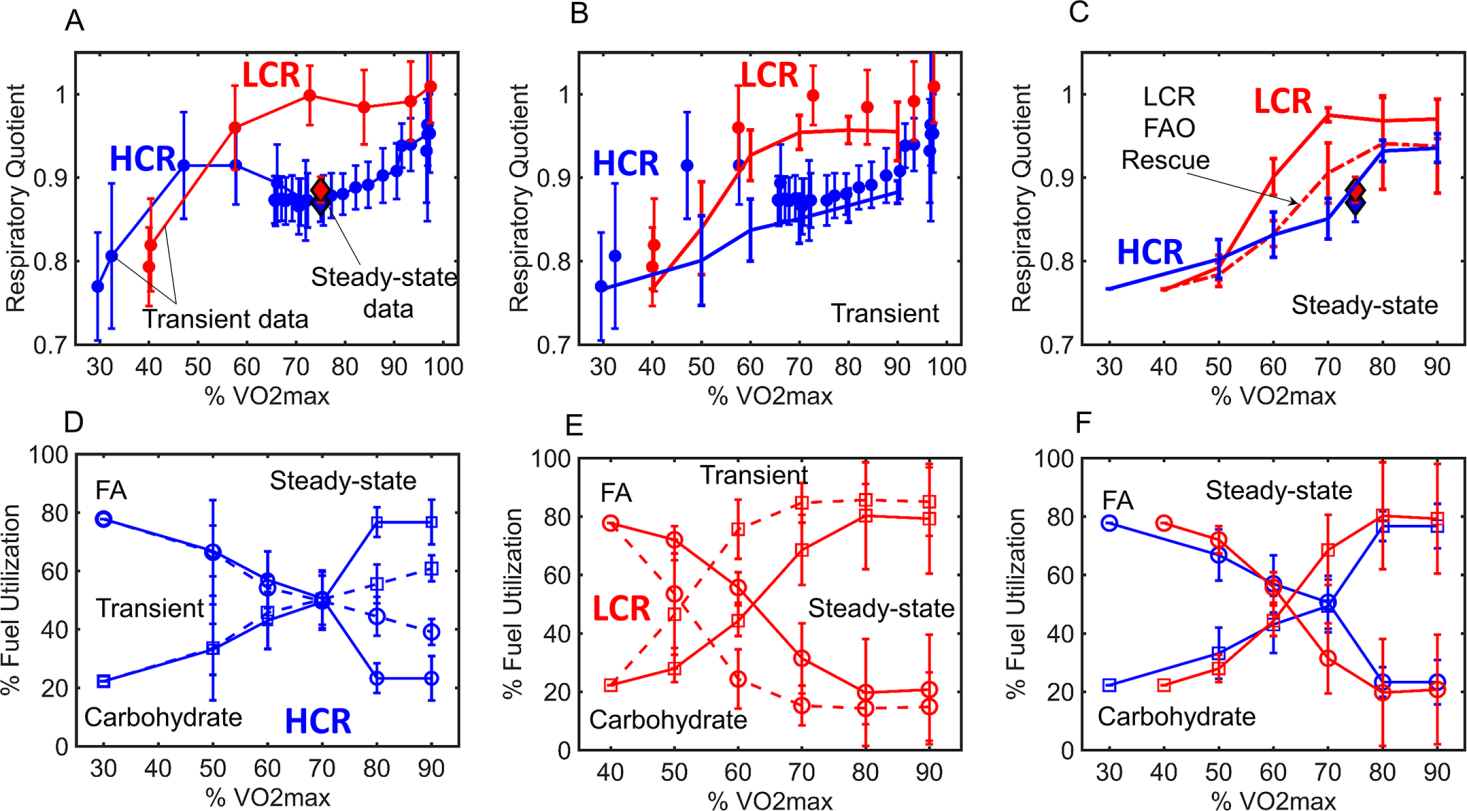
HCR and LCR transient and steady-state fuel utilization with normalized exercise. **(A) Data only:** Transient respiratory quotient (J_CO2_/J_O2_) data obtained from HCR (blue connected circles) and LCR (red connected circles) rats during the previously described treadmill exercise protocol [30] are plotted as a function of %VO2max. Steady-state respiratory quotient HCR (blue diamond) and LCR (red diamond) data obtained at 75% VO2max are also plotted in panel A. **(B) Transient data with transient simulations:** HCR and LCR parameter set families, transitioned from a resting state parameter set, were used to simulate transient normalized exercise intensity (described in Methods), as a function of %VO2max. HCR data are shown as blue circles (as in panel A) while the simulations are shown as blue lines. LCR data are shown as red circles (as in panel A) while the simulations are shown as red lines. Transient simulations were conducted by simulating exercise for 2 min at each %VO2max, as is the case with HCR and LCR transient data. **(C) Steady-state data with steady-state simulation:** Lines are simulated as in panel B except with 60 minutes of simulation time to mimic steady-state exercise data. HCR data are shown as blue diamonds while the HCR simulations are shown as blue lines. LCR data are shown as red diamonds while the LCR simulations are shown as red lines. LCR was also simulated without an additional reduction in FAO enzyme and FA transport activities (red dashed line), to better match the LCR steady-state data. **(D) Simulated HCR % fuel utilization:** HCR fuel utilization percentages with both transient (dashed lines) and steady-state (solid lines), normalized exercise intensity. FA (Fatty acid) percentages are shown as circles and carbohydrate are shown as squares. **(E) Simulated LCR % fuel utilization:** LCR fuel utilization percentages with both transient (dashed lines) and steady-state exercise (solid lines). FA percentages are shown as circles and carbohydrate are shown as squares. **(F) Simulated steady-state HCR and LCR %fuel utilization only:** Steady-state fuel utilization percentage estimates from panel D and E for HCR (blue) and LCR (red) are re-plotted in this panel for comparison.

To compare HCR and LCR fuel selection with normalized exercise intensity, we plotted the respiratory quotient (RQ) data for HCR and LCR, as a function of their %VO2max (Fig 13A). These data are the same as those shown in Fig 4D, with the addition of steady-state treadmill exercise data previously collected for both HCR and LCR at 75% VO2max (See supplemental data in [30]). The steady-state, RQ data at 75% VO2max (diamonds in Fig 13A) are different than the transient RQ data (circles in Fig 13A).The steady-state values were obtained by running HCR and LCR rats for a longer time period (data collected in the last 15 min of a 30 min run) at constant treadmill speed. In contrast, transient data were collected every 2 minutes, while treadmill speed increased every 2 minutes (circles in Fig 13A). Therefore, HCR and LCR rats have much less time to become acclimated to the exercise intensity in the transient exercise regimen, which increases in intensity every 2 minutes. It is noticeable from this plot that the transient LCR data (red circles) differ from the steady-state data (a red diamond) at 75% VO2max, but this is not the case for HCR (blue markers).

The model was simulated to calculate the RQ at various percentages of VO2max (Fig 13B) to match the transient (non-steady-state) data. Transient simulations were performed by simulating the model for 2 min at a constant ATPase work rate, and after each 2 min period the work rate was increased according to either HCR or LCR’s normalized work load (%VO2max). We discovered that when trying to simulate the model to long times that we needed to allow for enzyme activities to change between resting and exercise workloads to achieve steady-states to match the data in Fig 13. Thus, for the simulations in Fig 13, enzyme activities are transitioned from resting activities to exercise activities, as a function of %VO2max, using a monoexponential equation (Eq 5, Methods) with a transition constant (T_c_). A transition constant (T_c_) of 0.15 and 0.1 were used for HCR and LCR, respectively. A simulation time of 2 minutes was used to simulate the transient data (Fig 13B).

From here, we attempted to simulate the steady-state data at 75% VO2max (Fig 13C) by simply increasing the simulation time to 60 minutes. The LCR simulation does not match the steady-state data at 75% VO2max (Fig 13C; red solid line). Although the LCR steady-state simulation does not match the data, we could better simulate this data by omitting the additional decrease in FA catabolic enzyme activities (Fig 13C; red dashed line), which were needed to better match the transient LCR data (Figs 4 and 13B).

The predicted effect of time on fuel selection can be better seen by simulating the model and approximating the percent of FA and carbohydrate that are utilized as a function of %VO2max for HCR (Fig 13D) and LCR (Fig 13E). As the simulation time increases from 2 minutes (Fig 13D, dashed line connections) to 60 minutes (Fig 13D, solid line connections), only small changes are predicted for HCR. To simulate LCR steady-state fuel percentages (Fig 13E, solid line connections) we omitted the additional decrease in FA catabolic enzyme activities, since this change was needed to help match the LCR steady-state data (Fig 13C). Compared to HCR, we found that the difference between steady-state and transient simulations are more pronounced for LCR, especially at lower %VO2max (Fig 13E).

For comparison, simulated steady-state fuel percentages for HCR and LCR are shown in Fig 13F. The model predicts that in the steady-state at normalized exercise intensity (%VO2max) HCR and LCR have similar fuel selection profiles (Fig 13F). In other words, this analysis predicted the deficit in LCR FAO is transient and diminishes with sustained exercise.

## Discussion

HCR and LCR rats are models of two extremes of exercise aerobic capacity that differ primarily in skeletal muscle [29] fuel selection [30] and also demonstrate different susceptibilities to insulin resistance [36, 37]. Therefore, HCR and LCR rats provide an interesting means to study skeletal muscle metabolic remodeling, regarding fuel selection in exercise endurance, capacity, and metabolic disease.

Our main objective was to identify the minimal difference in enzyme activities between HCR and LCR rats, as they were subjected to graded treadmill exercise, to understand how HCR rats better sustain aerobic exercise compared to LCR rats. To accomplish this, we constructed a thermodynamically constrained model combining constraint-based analysis and ordinary differential equation modeling. We hypothesized that the model would show that LCR only differ from HCR by a moderate decrease in mitochondrial density (total mitochondrial activity). We also asked the question if a model completely based on mass-action kinetics and thermodynamic constraints, lacking detailed enzyme kinetic modeling, could still reproduce fuel switching dependent on ATP demand.

Constraint-based modeling, rooted in flux balance analysis [38], was first used to estimate the metabolic fluxes of a model explicitly accounting for glycolysis, the TCA cycle, mitochondrial electron transport reactions, and mitochondrial FA transport and β-oxidation (Figs 1 and 3) during HCR and LCR aerobic exercise (Fig 2). The estimation of these metabolic fluxes (Fig 3) allowed us to then estimate individual enzyme activities based on a simple generalized enzyme flux expression (Eq 3, Methods) and simulate the time-dependent data (Fig 4) in a minimal way. Our analysis revealed that to explain the minimal difference between HCR and LCR data, we needed to decrease both total mitochondrial enzyme activities (~50%) with, at least, an additional decrease in mitochondrial FA transport and oxidative activities (about an additional 5-fold decrease) (Fig 5).

It has been previously shown that LCR have similar mitochondrial density in red gastrocnemius muscle [34, 37], but are reduced in white [34], which corroborates the idea that LCR aerobic exercise capacity could be simply attenuated by a decrease in mitochondrial density. However, HCR and LCR muscle proteomic data during exercise [30] suggested additional differences. These experiments demonstrated significantly greater acetylation/phosphorylation of LCR FA transport and β-oxidative enzymes, among others, with respect to HCR during exercise [30]. These increases in posttranslational modification (PTM) are believed to decrease enzyme activities and hinder FA catabolism. Furthermore, HCR have also been shown to be enriched in oxidative phosphorylation and FA metabolism proteins [30, 37], thus enzyme expression levels also appear to be a factor.

Our model does not distinguish between enzyme activity effects induced by PTM or expression levels, however, it does support the need to invoke combined changes in both total mitochondrial activity (mitochondrial density) and a specific decrease in FA transport. Additional decreases in FA β-oxidation enzyme activities (Fig 5) were also invoked to better simulate LCR data, which are also supported by proteomics data [30]. According to model sensitivity analysis, enzymatic control over fuel selection during exercise differs between HCR and LCR due to HCR’s increased control over FA transport enzymes such as CPT1 and FAT (CD36) (Figs 9 and 10).

Our model is simplified in that it does not account for amino acid catabolism, although amino acid data were provided in the data set we analyzed [30]. This simplification is justified by our goal to gain insight into glucose and FA catabolism, which account for the bulk of the energy demand (85–90% [39]). However, the addition of amino acid catabolism could provide additional insight.

Despite the need to decrease HCR FA enzyme activities to simulate LCR data, it is important to understand that these data (Fig 4) were collected during a transient catabolic state. When these data are normalized for exercise intensity (%VO2max) and compared to steady-state data at 75% VO2max (Fig 13A) there is a discrepancy between the LCR transient and steady-state data. The steady-state respiratory quotients at 75% VO2max are similar between HCR and LCR (Fig 13A).

Thus, it appears that the difference between LCR and HCR FAO diminishes with sustained normalized exercise intensity (Fig 13). This result suggests that LCR may transition from attenuated fat catabolism during transient intense exercise to a more active fat catabolism when allowed longer time periods to acclimate to the exercise intensity. The transition between initial and steady-state activities may be achieved by PTM, as previously proposed [30]. Furthermore, the model also leads to the conclusion that FA transport is critical for fuel selection, recently demonstrated with CD36 KO and overexpression mice [27, 40].

Finally, it is interesting that the simple kinetic model developed here, for the HCR rat, can switch between FA and carbohydrate substrates in a physiological manner, as demonstrated by simulating the metabolic response to exercise. The model does not include allostery, such as required for the Randle cycle [20, 21] or calcium mediated activation of mitochondrial dehydrogenases. The model does not necessarily exclude these mechanisms as contributors to fuel selection *in vivo*, but does show that simple kinetic modeling can effectively capture this phenomenon without involving these regulatory processes.

## Methods

### Constraint-based solution

The computer model was initialized by building a stoichiometric matrix **S (**98 reactants by 87 reactions; Eq 1) accounting for all of the reactions shown in Fig 1, using previously developed computer code [32]. A full list of the reactions and their definitions can be found in S1 Table. As a first approximation of internal reaction fluxes, a steady-state approximation was implemented (Eq 1). In Eq 1, (***S***) is the stoichiometric matrix and ($\bar{J}$) is a vector of fluxes for each of the 87 reactions plus 6 transport fluxes.

$$S\cdot \bar{J}=\bar{0}$$ (1)

The transport fluxes are defined by the input of glucose, FA, O_2_, and the output of lactate, CO_2_, and H_2_O. Transport fluxes were determined using respiration and indirect calorimetry data shown in Fig 2 from HCR and LCR rats during a graded treadmill running exercise protocol [30]. Eq 1 was solved for ($\bar{J}$) using the FMINCON function in MATLAB 2016a, by applying *ab initio* thermodynamic cycle constraints [33], and maximizing mitochondrial ATP production as an objective function for each time point shown in Fig 2. Fig 3 shows an interpolated surface of the combined solutions of Eq 1 for HCR (Fig 3A) and LCR (Fig 3B) using the data in Fig 2 as transport fluxes at each time point.

### Thermodynamically feasible concentration vector

To obtain a thermodynamically feasible concentration vector needed to simulate the metabolic network (Fig 1), we first collected a standard transformed Gibbs free energy vector $\left(\Delta \bar{G^{\prime 0}}\right)$ for the 87 reactions in the network at 25°C and pH 7. Therefore, all reactions treat pH as a fixed entity throughout the course of the simulations. $\Delta \bar{G^{\prime 0}}$ was obtained primarily from the Goldberg [41], Li [42], and Alberty [43] databases, or calculated using the Equilibrator [44] server. $\Delta \bar{G^{\prime 0}}$ values can be found in S1 Table. A Monte Carlo method was applied to randomly choose metabolite concentrations that were consistent with literature concentration boundaries and satisfied Eq 2 (An expression of the 2^nd^ law of thermodynamics discussed in [45]), where *R* is the gas constant, *T* is absolute temperature, and $\bar{Q}$ is the mass action ratio vector.

$$\begin{array}{l} \Delta \bar{G^{\prime }}\cdot \bar{J}<0\\ \Delta \bar{G^{\prime }}=\Delta \bar{G^{\prime 0}}+RT\ln\bar{Q} \end{array}$$ (2)

The resulting concentration vector was slightly adjusted to match the data shown in Fig 4 for HCR and LCR. The final concentration vectors used to simulate HCR and LCR can be found in S2 Table.

### Model simulation

The model was simulated by solving the ordinary differential equation system in Eq 3, where (*C*) is the concentration vector, (***P***) is a partition matrix, (***S***) is the stoichiometric matrix, and (*J*_*calc*_) is the calculated reaction flux vector. The partition matrix is a diagonal matrix that compartmentalizes the system into extracellular, cytosolic, and mitochondrial spaces with partition coefficients of 0.2, 0.75, and 0.05, respectively, based on the fractional volume of skeletal muscle [46]. Here we have used skeletal muscle tissue spaces because this tissue type represents the dominant tissue for whole body metabolism during exercise. To account for the water volume of skeletal muscle, we used a value of 755 mL water per kg of muscle mass [46].

$$\frac{d\bar{C}}{dt}=P^{-1}S\cdot {\bar{J}}_{calc}$$ (3)

Reaction fluxes ($J_{calc}^{k}$) for each *k*^*th*^ reaction were calculated via a general flux expression (Eq 4) derived previously [32]. In Eq 4, (*X*) is the enzyme activity and (C_t_) is a concentration dependent term where *μ* and *ν* are the stoichiometry’s of the substrates and the products of reaction *k*, respectively. The C_t_ term is unitless due to individual terms being divided by the reference state of 1 M.

$$\begin{array}{l} J_{calc}^{k}=XC_{t}\left(\frac{1-e^{\frac{\Delta G^{\prime }}{RT}}}{1+e^{\frac{\Delta G^{\prime }-\Delta G^{\prime 0}}{RT}}}\right)\\ C_{t}^{k}=\prod_{i=1}^{m}{\left(C_{i}\right)}^{\mu i}+\prod_{i=1}^{m}{\left(C_{i}\right)}^{vi} \end{array}$$ (4)

The rate of ATP hydrolysis, required for work, throughout the exercise protocol was estimated by using the ATPase flux (Fig 3C) determined from the constraint-based solution for HCR. These determined fluxes were then fitted to a polynomial to estimate the ATPase rate at any time. This ATPase rate was then used as an input to drive the system and simulate work.

Model simulations were conducted using ode15s in MATLAB 2016a with an absolute tolerance of 10^−12^ and relative tolerance of 10^−10^, with the non-negative option on. Enzyme activities (*X* in Eq 4) were treated as constants throughout the course of the simulations for transient exercise data shown in Figs 4 and 7. However, long-term simulations, such as those shown in Fig 13, required transitioning the activities from resting activities (X_rest_) to exercise activities (X_exercise_). Activities were transitioned as a function of %VO2max using a general monoexponential equation (Eq 5) with a transition constant (T_c_).

$$X\left(\%VO2\max\right)=X_{exercise}+\left(X_{rest}-X_{exercise}\right)e^{\frac{-\left(\%VO2\max-\%VO2rest\right)}{T_{c}}}$$ (5)

Fuel utilization percentages, as shown in Fig 13D–13F, were calculated as follows: % FA utilization = (1-RQ)/(1–0.7); carbohydrate utilization = 100-% FA utilization; where RQ is the calculated respiratory quotient. Exercise enzyme activity parameters (X_exercise_) from 10^3^ parameter sets that were fitted to HCR and LCR data, and their corresponding sensitivity coefficients (ϕ), were used to calculate a normalized enzyme activity score to assess the greatest differences in enzyme activity while accounting for their sensitivity.

$$\begin{array}{l} {\underset{i}{X}}_{HCR}^{Score}=\left(\frac{{\underset{i}{X}}_{HCR}^{median}}{{\underset{i}{X}}_{LCR}^{median}}\right){\underset{i}{\phi }}_{{}_{HCR}}^{\begin{array}{l} median\\ sensitivity \end{array}}\\ {\underset{i}{X}}_{LCR}^{Score}=\left(\frac{{\underset{i}{X}}_{LCR}^{median}}{{\underset{i}{X}}_{HCR}^{median}}\right){\underset{i}{\phi }}_{{}_{LCR}}^{\begin{array}{l} median\\ sensitivity \end{array}} \end{array}$$ (6)

### Model parameterization and optimization

Enzyme activities (*X* in Eq 4) were treated as adjustable parameters when trying to fit the HCR data (blue) in Fig 4. Initial guesses for these activities were derived by solving for them algebraically by setting Eq 4 equal to fluxes from the constraint-based solution and plugging in the initial concentration vector. Simulations utilizing enzyme activities (*X* in Eq 4) derived using the initial resting fluxes from the constraint-based solution tended to fail prematurely at higher ATPase rates because these activities were too low. We arbitrarily increased enzyme activities to allow the simulations to withstand higher ATPase rates. From here, to fit HCR data, enzyme activities were adjusted to minimize the difference between the HCR data (Fig 4, blue) and the simulation using simulated annealing and Monte Carlo approaches, along with the FMINCON function in MATLAB 2016a. Error function values reported in the text were computed using Eq 7.

$$Error=\sqrt{\mathrm{mean}\left(\frac{{\left({\mathrm{data}}_{i}-{\mathrm{model}}_{i}\right)}^{2}}{{\mathrm{variance}}_{i}}\right)}$$ (7)

LCR parameters were obtained either by minimally changing HCR parameters (individual enzyme activities) to fit LCR data (Fig 4; red), or by allowing all enzyme activities to change to fit the LCR data (Fig 7; red). Total mitochondrial activity was adjusted from HCR to LCR by a multiplicative factor. For example, a 30% decrease in mitochondrial activity from HCR to LCR was defined as: X_LCR_(mito) = X_HCR_(mito)*(1–0.3). Further decreases in HCR activities, to achieve fitness to the LCR data, were explored by randomly adjusting different enzyme activities starting from small differences (Figs 4 and 5) with the goal of adjusting a minimal number of enzyme activity differences between model parameterizations representing the LCR versus the HCR data. With this minimal fitting approach, 10 LCR activity parameter sets were obtained starting from 10 HCR activity parameter sets. Additionally, a non-minimal approach to fitting LCR data (Fig 7; red) was implemented that allowed for all enzyme activities to change relative to HCR. This approach applied both global and local optimization methods to achieve fitness.

## Supporting information

S1 Table Enzyme reaction definitions, abbreviations, free energies, and activities. A spreadsheet containing all chemical reaction definitions, and the corresponding enzyme name and enzyme abbreviation, free energy of reaction, and enzyme activities used for simulation of main Fig 4.
(XLSX)

S2 Table Metabolite names (species), abbreviations, and concentrations. A spreadsheet containing all metabolites (species), an abbreviated species identifier where “_m” indicates mitochondrial, “_e” indicates extracellular, and no additional label indicates cytosolic species. Resting metabolite concentrations (initial conditions) are also given for HCR and LCR along with upper and lower boundaries (UB and LB, respectively) that were used in determining initial starting concentrations.
(XLSX)

S3 Table HCR metabolite exercise dependent (time dependent) concentrations. A spreadsheet containing molar (M) concentrations of all metabolites for HCR, based on the simulation shown in Fig 4 (blue solid line).
(XLSX)

S4 Table LCR metabolite exercise dependent (time dependent) concentrations. A spreadsheet containing molar (M) concentrations of all metabolites for LCR, based on the simulation shown in Fig 4 (red solid line).
(XLSX)

S1 Fig Maximum and minimum of one-thousand HCR constraint-based solutions. HCR constraint-based solutions of Eq 1 (in Methods) solved at each time point for internal reaction fluxes using given Carbohydrate, FA, O2, and CO2 transport fluxes. Transport fluxes were derived from HCR O2 and CO2 flux data shown in Fig 2. One-thousand solutions were produced from 1000 initial starting points of internal flux vectors spanning 18 orders of magnitude (10^−9^ to 10^9^). The maximum of these solutions are shown as red transparent mesh while the minimum of these solutions are shown as blue mesh.
(TIF)

S2 Fig Maximum and minimum of one-thousand LCR constraint-based solutions. LCR constraint-based solutions of Eq 1 (in Methods) solved at each time point for internal reaction fluxes using given Carbohydrate, FA, O2, and CO2 transport fluxes. Transport fluxes were derived from LCR O2 and CO2 flux data shown in Fig 2. One-thousand solutions were produced from 1000 initial starting points of internal flux vectors spanning 18 orders of magnitude (10^−9^ to 10^9^). The maximum of these solutions are shown as red transparent mesh while the minimum of these solutions are shown as blue mesh.
(TIF)

S3 Fig The ATPase (ATP → ADP + Pi) flux, maximum red circles and minimum blue circles, from the one-thousand constraint-based solutions using HCR transport fluxes. (TIF)

S4 Fig **A)** HCR and LCR metabolite concentrations determined from the simulation of transient exercise data described in main Fig 4 were used to plot a surface of metabolite ratios, [HCR (metabolites)]/[LCR (metabolites)] to diagnose differences in metabolite concentrations. The enzyme activities used for the simulation were from the best fitting hypothesis in Fig 4, blue solid line for HCR and red solid line for LCR. **B)** This panel is the same as in panel (A) except that it is the reciprocal to show [LCR (metabolites)]/[HCR (metabolites)], therefore metabolite concentrations that are greater in LCR become much more visible.
(TIF)

S1 Matlab Code A MATLAB file containing code to generate the plots associated with the figures. This code also contains the code required to simulate the model as is needed to generate several figures.
(ZIP)

S1 Matlab Figures MATLAB figures “.fig” files have been generated, and can be downloaded here, to visualize the figures without the need to run code that can take several minutes in some cases. (ZIP)
