# Supplementary figures and images for: Systems-level computational modeling demonstrates fuel selection switching in high capacity running and low capacity running rats
Source: PLoS Comput Biol. 2018 Feb 23;14(2):e1005982. doi: 10.1371/journal.pcbi.1005982 (PMC5841818; doi:10.1371/journal.pcbi.1005982)

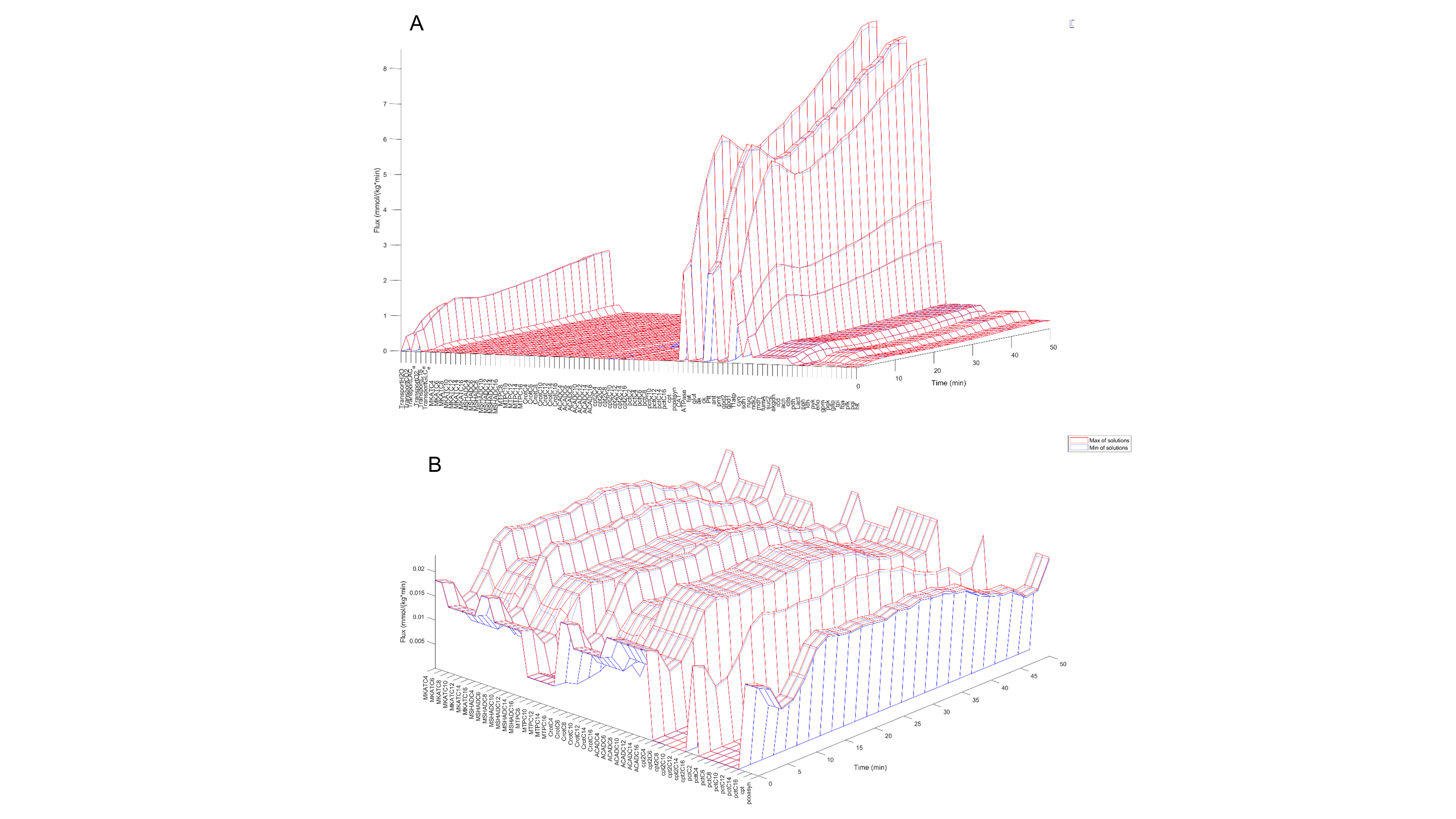

Supplement: S1 Fig — HCR constraint-based solutions of Eq 1 (in Methods) solved at each time point for internal reaction fluxes using given Carbohydrate, FA, O2, and CO2 transport fluxes. Transport fluxes were derived from HCR O2 and CO2 flux data shown in Fig 2. One-thousand solutions were produced from 1000 initial starting points of internal flux vectors spanning 18 orders of magnitude (10−9 to 109). The maximum of these solutions are shown as red transparent mesh while the minimum of these solutions are shown as blue mesh. (TIF) [file pcbi.1005982.s005.tif]

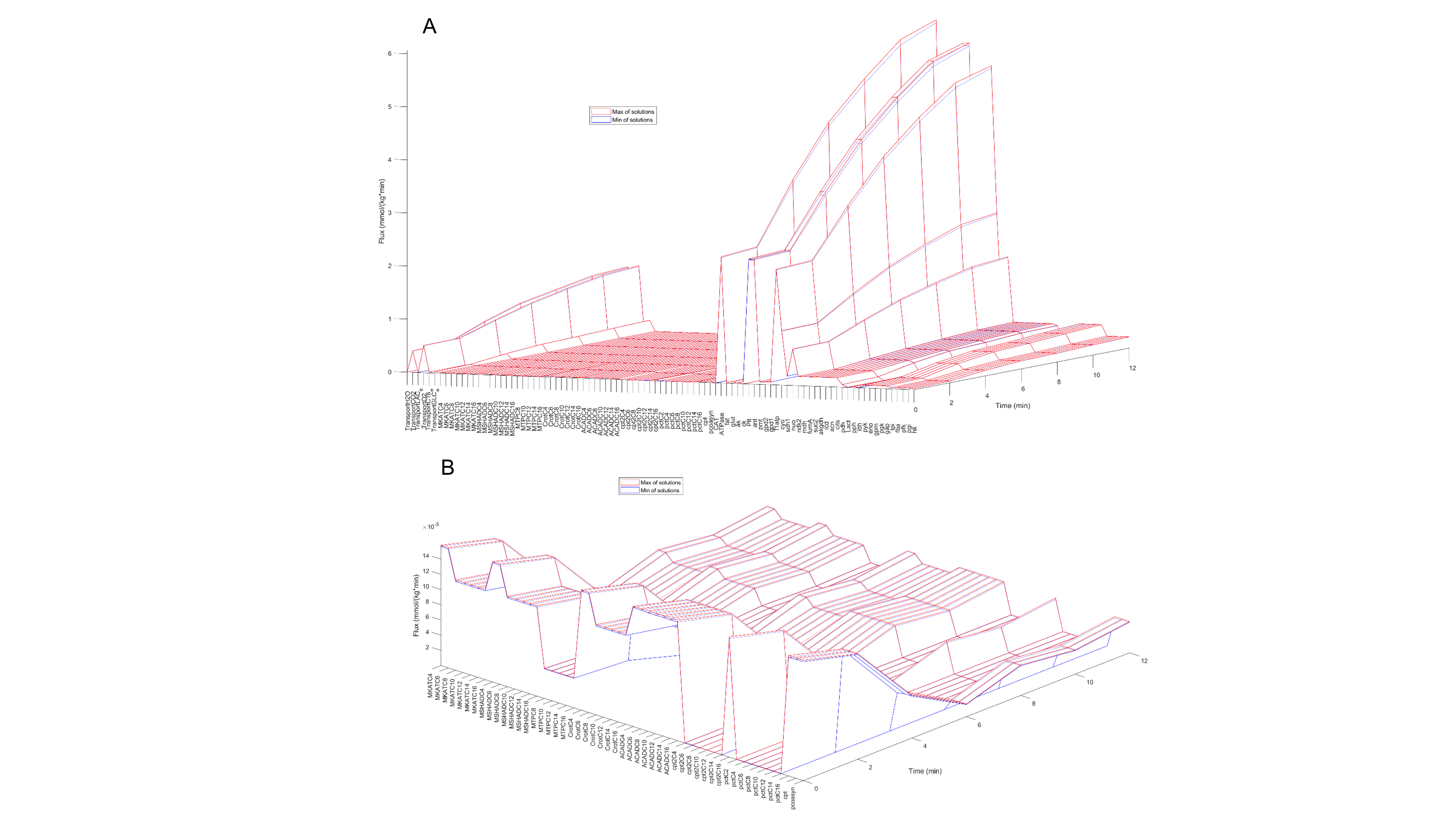

Supplement: S2 Fig — LCR constraint-based solutions of Eq 1 (in Methods) solved at each time point for internal reaction fluxes using given Carbohydrate, FA, O2, and CO2 transport fluxes. Transport fluxes were derived from LCR O2 and CO2 flux data shown in Fig 2. One-thousand solutions were produced from 1000 initial starting points of internal flux vectors spanning 18 orders of magnitude (10−9 to 109). The maximum of these solutions are shown as red transparent mesh while the minimum of these solutions are shown as blue mesh. (TIF) [file pcbi.1005982.s006.tif]

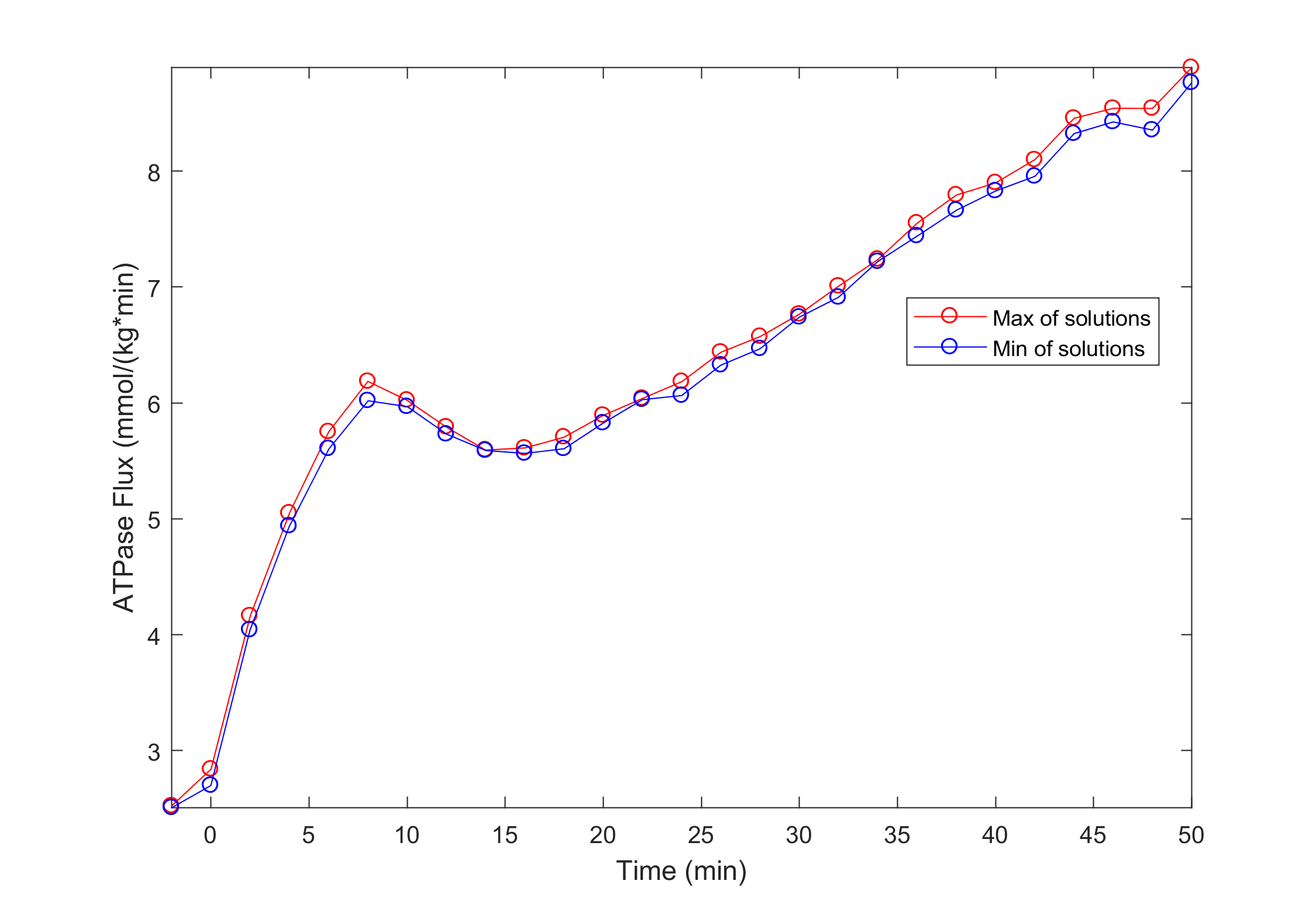

Supplement: S3 Fig — (TIF) [file pcbi.1005982.s007.tif]

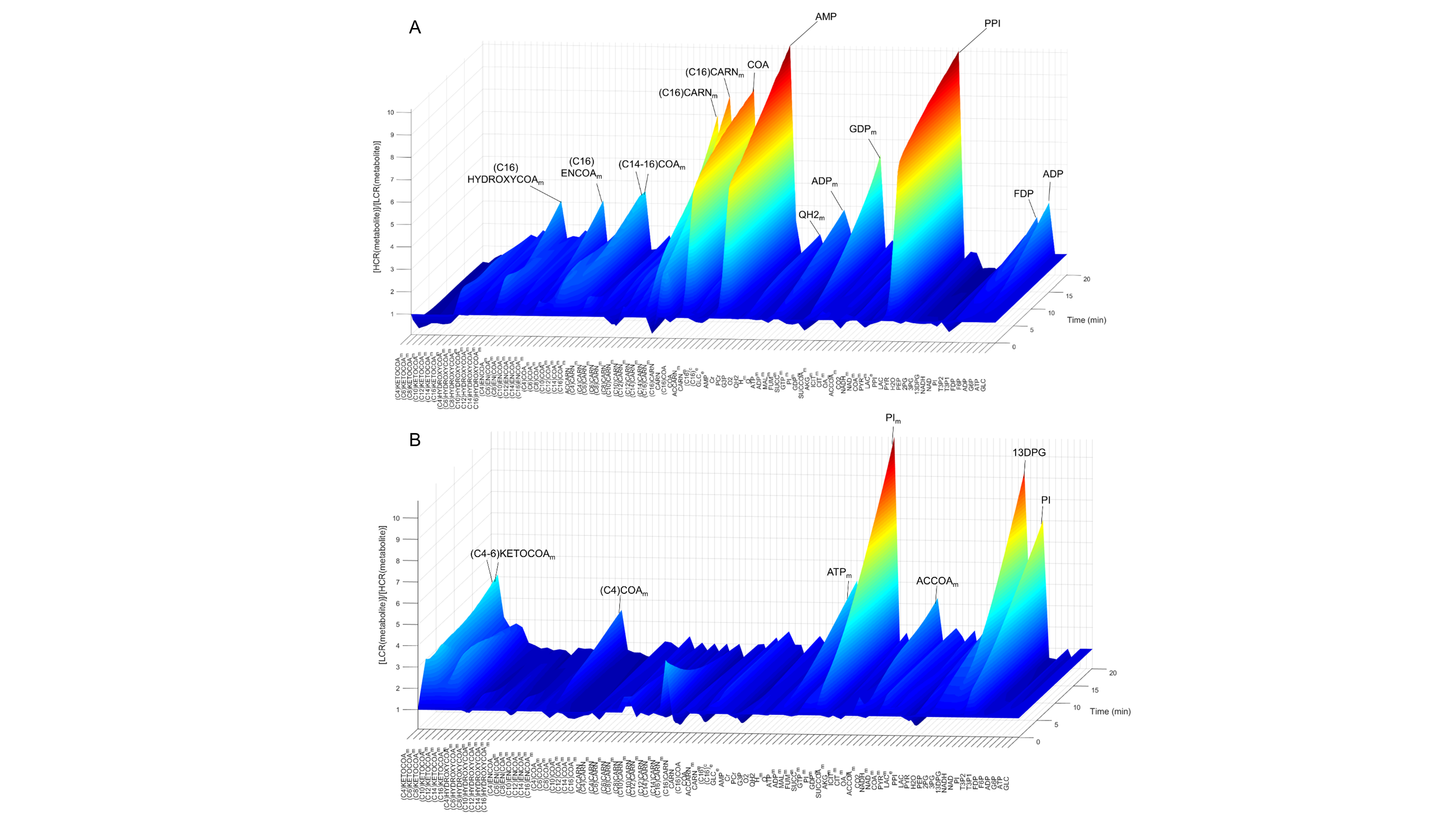

Supplement: S4 Fig — A) HCR and LCR metabolite concentrations determined from the simulation of transient exercise data described in main Fig 4 were used to plot a surface of metabolite ratios, [HCR (metabolites)]/[LCR (metabolites)] to diagnose differences in metabolite concentrations. The enzyme activities used for the simulation were from the best fitting hypothesis in Fig 4, blue solid line for HCR and red solid line for LCR. B) This panel is the same as in panel (A) except that it is the reciprocal to show [LCR (metabolites)]/[HCR (metabolites)], therefore metabolite concentrations that are greater in LCR become much more visible. (TIF) [file pcbi.1005982.s008.tif]
